# Highly and Broad-Spectrum *In Vitro* Antitumor Active *cis*-Dichloridoplatinum(II) Complexes with 7-Azaindoles

**DOI:** 10.1371/journal.pone.0136338

**Published:** 2015-08-26

**Authors:** Pavel Štarha, Zdeněk Dvořák, Zdeněk Trávníček

**Affiliations:** Regional Centre of Advanced Technologies, Division of Biologically Active Complexes and Molecular Magnets, Faculty of Science, Palacký University, Olomouc, Czech Republic; Virginia Commonwealth University, UNITED STATES

## Abstract

The *cis*-[PtCl_2_(*n*aza)_2_] complexes (**1**–**3**) containing monosubstituted 7-azaindole halogeno-derivatives (*n*aza), showed significantly higher activity than *cisplatin* towards ovarian carcinoma A2780, its *cisplatin*-resistant variant A2780R, osteosarcoma HOS, breast carcinoma MCF7 and cervix carcinoma HeLa cell lines, with the IC_50_ values of 3.8, 3.5, 4.5, 2.7, and 9.2 μM, respectively, obtained for the most active complex **3**. As for **4** and **5** having disubstituted 7-azaindoles in their molecule, the significant cytotoxicity was detected only for **4** against A2780 (IC_50_ = 4.8 μM), A2780R (IC_50_ = 3.8 μM) and HOS (IC_50_ = 4.3 μM), while **5** was evaluated as having only moderate antiproliferative effect against the mentioned cancer cell lines with IC_50_ = 33.4, 24.7 and 46.7 μM, respectively. All the studied complexes **1**–**5** effectively avoided the acquired resistance of ovarian carcinoma cell line. On the other hand, the complexes did not reveal any inhibition activity on the purified 20S proteasome from the A2780 cells. The representative complexes **3** and **5** showed low ability to be hydrolysed, but their stability was markedly lowered in the presence of physiological sulphur-containing biomolecule glutathione (GSH), as proved by the ^1^H NMR spectroscopy and mass spectrometry studies. A rate of interaction of the studied complexes with GSH was affected by an addition of another mechanistically relevant biomolecule guanosine monophosphate. The differences in interactions of **3** and **5** with GSH correlate well with their different cytotoxicity profiles.

## Introduction

A clinically successful metal-based anticancer drug *cisplatin*, *cis*-[Pt(NH_3_)_2_Cl_2_] [1,2], was followed by a lot of platinum complexes, which entered or even completed the clinical trials with the aim to become an improved *cisplatin* analogue in the treatment of cancer diseases [3,4]. Interestingly, the general structural characterization of the mononuclear platinum(II) analogues of *cisplatin* is quite strict and can be generalized as follows: 1/ the leaving groups are either two chloride anions (*e*.*g*. *NSC 170898* [5] or *picoplatin* [6]) or one bidentate (*e*.*g*. world-wide clinically used *carboplatin* [7] or *oxaliplatin* [8]) or two monodentate (*aroplatin* [9]) carboxylates, with an exception of *spiroplatin* [10] and *ProLindac* [11]; 2/ the carrier ligands are either two ammines (*e*.*g*. *carboplatin*) or one bidentate (*e*.*g*. *oxaliplatin*) or two monodentate (*e*.*g*. *NSC 170898*) amines; *NSC 170898* = *cis*-dichlorido-bis(cyclopentylamine)platinum(II), *picoplatin* = *cis*-ammine-dichlorido-(2-methylpyridine)platinum(II), *carboplatin =* diammine-(cyclobutane-1,1-dicarboxylato)platinum(II), *oxaliplatin* = 1*R*,2*R*-cyclohexanediamineoxalatoplatinum(II), *aroplatin* = 1*R*,2*R*-diaminocyclohexane-bis(neodecanaoto)platinum(II), *spiroplatin* = cyclohexane-1,1-dimethylamine-sulfatoplatinum(II), *ProLindac* = a polymeric prodrug of 1*R*,2*R*-cyclohexanediamineplatinum(II) bound to hydroxypropylmethacrylamide. As for the carrier ligands, there can be also found the exceptions, namely the mentioned *picoplatin* or *miboplatin* (*R*-2-aminomethylpyrrolidine-(cyclobutane-1,1-dicarboxylato)platinum(II)), which involve the heterocyclic *N*-donor carrier ligands [12]. The mentioned *picoplatin* involving, in comparison with *cisplatin*, one bulky 2-methylpyridine *N*-donor ligand instead of one ammine, showed very promising results on a wide spectrum of tumour types including those resistant to *cisplatin* and even *oxaliplatin* [13,14]. Introducing of the mentioned 2-methylpyridine into the structure of *picoplatin* results in slower hydrolysis rate and different p*K*_a_ values as compared with *cisplatin* [15], and causes the steric hindrance of the Pt(II) atom which consequently hinders approach of nucleophiles (*e*.*g*. glutathione) to the Pt(II) atom. *Picoplatin* failed in the Phase II clinical trials against both small and non-small cell lung cancer, but it is currently investigated against colorectal and prostate cancers.

The pharmacological perspective of the platinum(II) complexes involving *N*-donor heterocyclic ligands can be also highlighted by a very recently published monofunctional platinum(II) complex *phenanthriplatin* (*cis*-diammine-chlorido-phenanthridineplatinum(II) nitrate) [16,17]. This compound, which monofunctionally interacts with DNA and more efficiently binds nucleobases than sulphur-containing biomolecules (5′-deoxyguanosine monophosphate and *N*-acetyl methionine were used as the model systems), showed extraordinary anticancer effectivity and no correlation with any other platinum-based anticancer drug at NCI-60 DTP Human Tumor Cell Line Screen, which favours *phenanthriplatin* for the future clinical testing.

In our laboratory, we deal with platinum(II) complexes involving various *N*-donor heterocyclic ligands, such as *N*6-benzyladenine or 7-azaindole derivatives (*e*.*g*. [18,19]), for more than ten years. In the case of the latter ones, we recently reported a series of *cis*-dichloridoplatinum(II) complexes involving 3-chloro-7-azaindole (*3Cl*aza), 3-iodo-7-azaindole (*3I*aza) and 5-bromo-7-azaindole (*5Br*aza) together with their significant *in vitro* cytotoxicity against a panel of human cancer cell lines, mechanistic studies and promising *in vivo* anticancer activity on the mouse model of L1210 lymphocytic leukaemia [19–21]. Some details regarding the mentioned biological aspects will be discussed below within the present paper.

**Fig 1:**
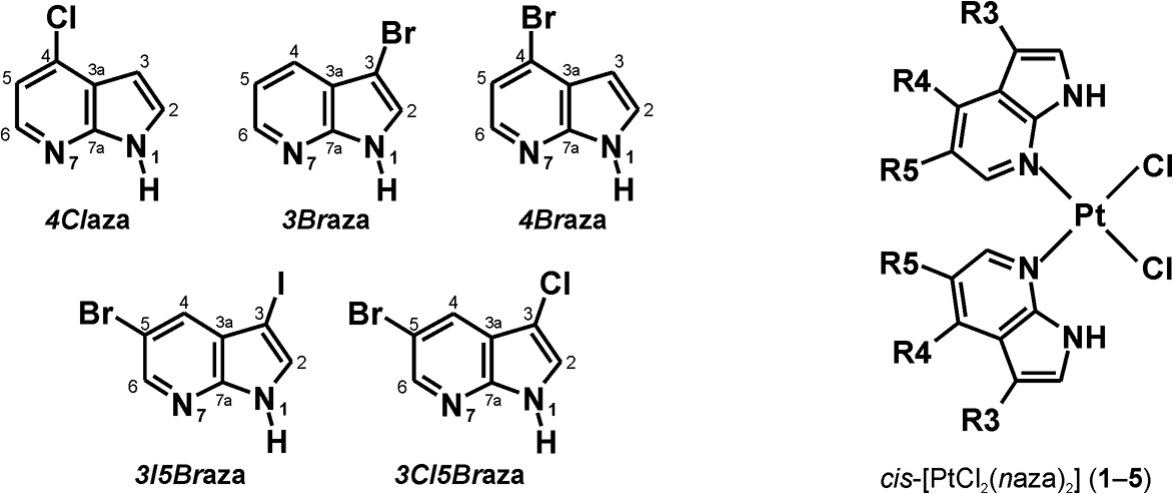
Structural formula of the studied complexes *cis*-[PtCl_2_(*n*aza)_2_] (1–5) and 7-azaindole derivatives used for their preparation. The general structural formulas of used 4-chloro-7-azaindole (*4Cl*aza; complex **1**), 3-bromo-7-azaindole (*3Br*aza; **2**), 4-bromo-7-azaindole (*4Br*aza; **3**), 3-iodo-5-bromo-7-azaindole (*3I5Br*aza; **4**) and 3-chloro-5-bromo-7-azaindole (*3Cl5Br*aza; **5**) are given together with their atom numbering scheme.

Herein we report a new series of the *cis*-dichloridoplatinum(II) complexes containing halogeno-derivatives of 7-azaindole different from the above mentioned ones (namely 4-chloro-7-azaindole (*4Cl*aza), 3-bromo-7-azaindole (*3Br*aza), 4-bromo-7-azaindole (*4Br*aza), 3-iodo-5-bromo-7-azaindole (*3I5Br*aza) and 3-chloro-5-bromo-7-azaindole (*3Cl5Br*aza); Fig 1). The complexes were prepared and studied with an aim to clarify an effect of the type (chloro, bromo and iodo), position (3, 4 and 5) and rate (mono- and disubstituted derivatives) of the 7-azaindole ring derivatization by the mentioned halogens to the resulting *in vitro* cytotoxicity. Additionally to our recently reported studies of the analogical complexes with differently substituted 7-azaindole moiety [19–21], we 1) studied more deeply the interaction with the biomolecules relevant from the mechanistic point of view (reduced glutathione (GSH) and guanosine 5'-monophosphate disodium salt hydrate (GMP)) on the selected representative complexes **3** (highly *in vitro* cytotoxic) and **5** (moderately *in vitro* cytotoxic), and 2) research the inhibition activity of the described complexes on the purified 20S proteasome extracted from the A2780 human ovarian carcinoma cell line.

## Materials and Methods

### Chemicals

The chemicals (K_2_[PtCl_4_], 4-chloro-7-azaindole (*4Cl*aza), 3-bromo-7-azaindole (*3Br*aza), 4-bromo-7-azaindole (*4Br*aza), 3-iodo-5-bromo-7-azaindole (*3I5Br*aza), 3-chloro-5-bromo-7-azaindole (*3Cl5Br*aza), *cisplatin*, reduced glutathione (GSH), guanosine 5'-monophosphate disodium salt hydrate (GMP)) and solvents (ethanol, *N*,*N`*-dimethylformamide, acetone, methanol, diethyl ether) were purchased from Sigma-Aldrich (Prague, Czech Republic) and Acros Organics (Pardubice, Czech Republic).

### Synthesis

Complexes were prepared as recently described for their analogues containing differently substituted 7-azaindole derivatives [19]. Briefly, the water solution of 0.5 mmol of K_2_[PtCl_4_] was mixed together with the ethanolic solution of 1.0 mmol of the appropriate 7-azaindole derivative (*4Cl*aza for **1**, *3Br*aza for **2**, *4Br*aza for **3**, *3I5Br*aza for **4** and *3Cl5Br*aza for **5**) (Fig 1). The products, which formed during 24 h of stirring at 50°C, were filtered off, washed (2 × 5 mL of distilled water, 2 × 5 mL of ethanol and 2 × 5 mL of diethyl ether), dried under vacuum and stored without any further purification.

*cis*-[PtCl_2_(*4Cl*aza)_2_] (**1**): *Anal*. Calcd. for C_14_H_10_N_4_Cl_4_Pt: C, 29.44; H, 1.76; N, 9.81%; found: C, 29.39; H, 1.79; N, 9.80%. ^1^H NMR (DMF-*d*_*7*_, ppm): δ 13.52 (br, N1H, 1H), 8.88 (d, 6.2, C6H, 1H), 7.94 (t, 3.2, C2H, 1H), 7.32 (d, 5.9, C5H, 1H), 6.67 (m, C3H, 1H). ^13^C NMR (DMF-*d*_*7*_, ppm): δ 147.8 (C7a), 145.8 (C6), 137.7 (C4), 128.9 (C2), 122.1 (C3a), 117.3 (C5), 100.2 (C3). ^15^N NMR (DMF-*d*_*7*_, ppm): δ 145.8 (N1), 168.0 (N7). ^195^Pt NMR (DMF-*d*_*7*_, ppm): δ −2113.2. ESI+ MS (methanol, *m/z*): 609.0 (calc. 608.9; 30%; {[PtCl_2_(*4Cl*aza)_2_]+K}^+^), 593.1 (calc. 592.9; 10%; {[PtCl_2_(*4Cl*aza)_2_]+Na}^+^), 153.1 (calc. 153.0; 5%; {*4Cl*aza+H}^+^). ESI–MS (methanol, *m/z*): 568.8 (calc. 568.9; 15%; {[PtCl_2_(*4Cl*aza)_2_]–H}^–^, 417.1 (calc. 416.9; 100%; {[PtCl_2_(*4Cl*aza)]–H}^–^), 151.2 (calc. 151.0; 10%; {*4Cl*aza–H}^–^).

*cis*-[PtCl_2_(*3Br*aza)_2_] (**2**): *Anal*. Calcd. for C_14_H_10_N_4_Br_2_Cl_2_Pt: C, 25.48; H, 1.53; N, 8.49%; found: C, 25.28; H, 1.53; N, 8.63%. ^1^H NMR (DMF-*d*_*7*_, ppm): δ 13.54 (br, N1H, 1H), 9.02 (d, 5.8, C6H, 1H), 8.10 (d, 2.3, C2H, 1H), 7.96 (d, 8.0, C4H, 1H), 7.30 (m, C5H, 1H). ^13^C NMR (DMF-*d*_*7*_, ppm): δ 146.7 (C6), 146.5 (C7a), 129.9 (C2), 127.6 (C4), 122.4 (C3a), 117.8 (C5), 89.0 (C3). ^15^N NMR (DMF-*d*_*7*_, ppm): δ 143.9 (N1), 172.8 (N7). ^195^Pt NMR (DMF-*d*_*7*_, ppm): δ −2126.2. ESI+ MS (methanol, *m/z*): 698.8 (calc. 698.8; 100%; {[PtCl_2_(*3Br*aza)_2_]+K}^+^), 625.0 (calc. 624.9; 20%; [PtCl(*3Br*aza)_2_]^+^), 197.1 (calc. 197.0; 5%; {*3Br*aza+H}^+^). ESI–MS (methanol, *m/z*): 658.9 (calc. 658.8; 100%; {[PtCl_2_(*3Br*aza)_2_]–H}^–^), 461.1 (calc. 460.9; 50%; {[PtCl_2_(*3Br*aza)]–H^–^}), 195.1 (calc. 195.0; 10%; {*3Br*aza–H}^–^).

*cis*-[PtCl_2_(*4Br*aza)_2_] (**3**): *Anal*. Calcd. for C_14_H_10_N_4_Br_2_Cl_2_Pt: C, 25.48; H, 1.53; N, 8.49%; found: C, 25.20; H, 1.59; N, 8.01%. ^1^H NMR (DMF-*d*_*7*_, ppm): δ 13.53 (s, N1H, 1H), 8.79 (d, 6.5, C6H, 1H), 7.96 (t, 3.3, C2H, 1H), 7.46 (d, 6.3, C5H, 1H), 6.60 (m, C3H, 1H). ^13^C NMR (DMF-*d*_*7*_, ppm): δ 146.9 (C7a), 145.5 (C6), 128.9 (C2), 127.2 (C3a), 124.5 (C4), 120.4 (C5), 101.8 (C3). ^15^N NMR (DMF-*d*_*7*_, ppm): δ 145.8 (N1), 168.4 (N7). ^195^Pt NMR (DMF-*d*_*7*_, ppm): δ −2116.2. ESI+ MS (methanol, *m/z*): 698.7 (calc. 698.8; 10%; {[PtCl_2_(*4Br*aza)_2_]+K}^+^), 682.8 (calc. 682.8; 20%; {[PtCl_2_(*4Br*aza)_2_]+Na}^+^), 197.0 (calc. 197.0; 5%; {*4Br*aza+H}^+^). ESI–MS (methanol, *m/z*): 658.7 (calc. 658.8; 35%; {[PtCl_2_(*4Br*aza)_2_]–H}^–^), 460.9 (calc. 460.9; 50%; {[PtCl_2_(*3Br*aza)]–H}^–^).

*cis*-[PtCl_2_(*3I5Br*aza)_2_] (**4**): *Anal*. Calcd. for C_14_H_8_N_4_Br_2_Cl_2_I_2_Pt: C, 18.44; H, 0.88; N, 6.14%; found: C, 18.29; H, 1.01; N, 6.17%. ^1^H NMR (DMF-*d*_*7*_, ppm): δ 13.88 (br, N1H, 1H), 9.34 (s, C6H, 1H), 8.17 (d, 2.5, C4H, 1H), 7.99 (s, C2H, 1H). ^13^C NMR (DMF-*d*_*7*_, ppm): δ 146.7 (C7a), 146.7 (C6), 134.4 (C4), 133.7 (C2), 126.9 (C3a), 111.1 (C5), 55.1 (C3). ^15^N NMR (DMF-*d*_*7*_, ppm): δ 150.1 (N1), 177.4 (N7). ^195^Pt NMR (DMF-*d*_*7*_, ppm): δ −2117.7. ESI+ MS (methanol, *m/z*): 950.5 (calc. 950.6; 30%; {[PtCl_2_(*3I5Br*aza)_2_]+K}^+^), 323.1 (calc. 322.9; 35%; {*3I5Br*aza+H}^+^). ESI–MS (methanol, *m/z*): 910.5 (calc. 910.6; 60%; {[PtCl_2_(*3I5Br*aza)_2_]–H}^–^), 587.0 (calc. 586.8; 100%; {[PtCl_2_(*3I5Br*aza)]–H}^–^), 321.1 (calc. 320.9; 10%; {*3I5Br*aza–H}^–^).

*cis*-[PtCl_2_(*3Cl5Br*aza)_2_] (**5**): *Anal*. Calcd. for C_14_H_8_N_4_Br_2_Cl_4_Pt: C, 23.07; H, 1.11; N, 7.69%; found: C, 23.12; H, 1.15; N, 7.83%. ^1^H NMR (DMF-*d*_*7*_, ppm): δ 13.37 (s, N1H, 1H), 8.91 (d, 1.9, C6H, 1H), 8.45 (d, 1.9, C4H, 1H), 8.15 (d, 2.8, C2H, 1H). ^13^C NMR (DMF-*d*_*7*_, ppm): δ 149.5 (C6), 144.7 (C7a), 131.4 (C4), 127.2 (C2), 121.8 (C3a), 110.3 (C5), 103.7 (C3). ^15^N NMR (DMF-*d*_*7*_, ppm): δ 142.4 (N1), 165.9 (N7). ^195^Pt NMR (DMF-*d*_*7*_, ppm): δ −2183.0. ESI+ MS (methanol, *m/z*): 231.1 (calc. 230.9; 70%; {*3Cl5Br*aza+H}^+^). ESI–MS (methanol, *m/z*): 496.7 (calc. 496.8; 40%; {[PtCl_2_(*3Cl5Br*aza)]–H}^–^).

### Physical Measurements

Elemental analysis (C, H, N) was performed on a Flash 2000 CHNS Elemental Analyzer (Thermo Scientific). Electrospray ionization (ESI) mass spectra (methanol solutions) were obtained by an LCQ Fleet ion trap spectrometer (Thermo Scientific; QualBrowser software, version 2.0.7) in both positive (ESI+) and negative (ESI–) ionization modes. ^1^H, ^13^C and ^195^Pt NMR spectra and ^1^H–^1^H gs-COSY, ^1^H–^13^C gs-HMQC, ^1^H–^13^C gs-HMBC and ^1^H–^15^N gs-HMBC two dimensional correlation experiments (gs = gradient selected, COSY = correlation spectroscopy, HMQC = heteronuclear multiple quantum coherence, HMBC = heteronuclear multiple bond coherence) of the DMF-*d*_*7*_ solutions were measured at 300 K on a Varian 400 device at 400.00 MHz (^1^H), 100.58 MHz (^13^C), 86.00 MHz (^195^Pt) and 40.53 MHz (^15^N). ^1^H and ^13^C NMR spectra were calibrated against the residual DMF-*d*_*6*_^1^H NMR (8.03, 2.92 and 2.75 ppm) and ^13^C NMR (163.15, 34.89 and 29.76 ppm) signals. ^195^Pt spectra were adjusted against K_2_[PtCl_6_] in D_2_O found at 0 ppm. ^1^H–^15^N gs-HMBC experiments were obtained at natural abundance and calibrated against the residual signals of DMF adjusted to 8.03 ppm (^1^H) and 104.7 ppm (^15^N). The splitting of proton resonances in the reported ^1^H spectra is defined as s = singlet, d = doublet, t = triplet, br = broad band, m = multiplet. Infrared spectra (150–600 cm^–1^ and 400–4000 cm^–1^ regions) were recorded on a Nexus 670 FT-IR (Thermo Nicolet) using the ATR technique. Raman spectra (150 and 3750 cm^–1^; except for **3** and **5**, which burnt under laser beam) were recorded by an NXR FT-Raman Module (Thermo Nicolet).

#### Studies of stability and interaction with biomolecules

The representative complexes **3** and **5** were dissolved in the DMF-*d*_*7*_/D_2_O mixture (1:1 *v/v*) and their ^1^H NMR spectra were recorded on the fresh solution (0 h) and after 24 h and 48 h of standing at laboratory temperature. The same ^1^H NMR experiments, but with an addition of two molar equivalents of GSH (the experiments and spectra labeled as **3**+GSH and **5**+GSH), GMP (**3**+GMP and **5**+GMP) or GSH/GMP mixture (**3**+GSH/GMP and **5**+GSH/GMP) were carried out as well.

Complex **3** (10 μM final concentration) or the mixture of **3** (10 μM final concentration) with GSH (6 μM final concentration [22]), both dissolved in the methanol/water mixture (1:1 *v/v*) were analysed by flow-injection analysis ESI-MS in both the positive and negative ionization mode immediately after the preparation (fresh solution) and after 2 and 24 h of standing at laboratory temperature. The mobile phase (90% methanol and 10% of 10 mM ammonium acetate) was pumped (0.2 mL/min) by the quaternary pump of Dionex Ultimate 3000 HPLC System. The samples were injected directly into the mobile phase flow (HPLC autosampler).

### Biological Studies

#### Cell culture and *in vitro* cytotoxicity testing

*In vitro* cytotoxic effect of **1**–**5** and *cisplatin* was assessed by an MTT assay [MTT = 3-(4,5-dimethylthiazol-2-yl)-2,5-diphenyl-tetrazolium bromide] against A2780 ovarian carcinoma, A2780R *cisplatin*-resistant ovarian carcinoma, HOS osteosarcoma, G361 malignant melanoma, MCF7 breast carcinoma, A549 lung carcinoma, HeLa cervix epithelia carcinoma and LNCaP prostate carcinoma human cancer cell lines, which were purchased from European Collection of Cell Cultures (ECACC). The cell lines were cultured according to the ECACC instructions and they were maintained at 37°C and 5% CO_2_ in a humidified incubator. The cells were treated with the solutions of **1**–**5** and *cisplatin* for 24 h, using multi-well culture plates of 96 wells. In parallel, the cells were treated with vehicle (DMF; 0.1%, v/v) and Triton X-100 (1%, v/v) to assess the minimal (100% viability) and maximal (0% viability) cell damage, respectively. The MTT assay was measured spectrophotometrically at 540 nm (TECAN, Schoeller Instruments LLC).

Analogical testing was carried out for **3** towards A2780 cancer cells with addition of L-buthionine sulfoximine (L-BSO). L-BSO was independently added to each well to give the 5.0 μM final concentration known to be non-toxic and optimal for the experiments focusing on the modulation of anticancer activity. The experiments with L-BSO were performed with two negative controls (DMF and 5.0 μM L-BSO) with no statistically different results obtained between both the controls.

The data from the cancer cells were acquired from three independent experiments (conducted in triplicate) using cells from three consecutive passages. The resulting IC_50_ values (μM) were calculated from viability curves and the results are presented as arithmetic mean±SD. The significance of the differences between the obtained results (*p* < 0.05 considered to be significant) was assessed by the ANOVA analysis (QC Expert 3.2, Statistical software, TriloByte Ltd.).

#### Inhibition of proteasome activity in purified 20S proteasome

A2780 cancer cell line was cultured in the complete RPMI 1640 medium (Sigma-Aldrich), supplemented with 10% heat-inactivated fetal-bovine serum (Sigma-Aldrich), 5 mL penicillin/streptomycin solution (PAA-cell culture company) and 2 mM L-glutamine (Sigma-Aldrich) at 37°C in a 5% CO_2_ atmosphere. Cells were passaged accordingly. The purification protocol was based upon previously published methods adapted for purification of the proteasomes from various tissues [23,24]. Cells were washed with 2 mL of ice-cold PBS 1x, scrapped and lysed by sonication in buffer (100 mM Tris, 5 mM MgCl_2_, 5 mM ATP, 0.5 mM DTT, 20% glycerol (pH 7.8)). The soluble protein fraction was isolated by centrifugation at 20,000 g for 30 min at 4°C. The pellet was discarded, and the supernatant fraction was centrifuged again at 100,000 g for 6 h at 4°C. The supernatant fraction was discarded, and the pellet was washed carefully in fresh buffer (without glycerol), which was also discarded. The washed pellets were re-suspended in 1 mL of buffer (per pellet) and stored at -80°C in 0.1 mL aliquots. The chymotrypsin-like activity of purified 20S proteasome was determined as follows: 5 μg of purified proteasome was pre-incubated with or without compounds for 20 min in 90 μL of assay buffer [30 mm Tris-HCl (pH 8.0)] at 37°C, followed by the incubation with 20 μm of fluorogenic peptide substrate (Suc-LLVY-AMC) (Sigma-Aldrich), at 37°C for 1 h. After the incubation, the fluorescence of the hydrolysed (AMC) groups in reaction mixtures was measured at 380/460 nm (TECAN, Infinite M200PRO).

## Results

### Chemistry

**Fig 2:**
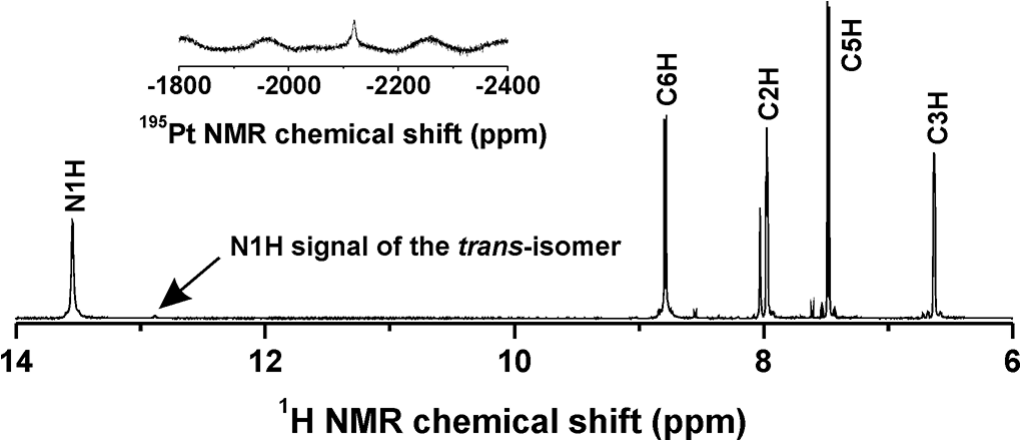
The ^1^H NMR and ^195^Pt NMR (inset) spectra of 3 in DMF-*d*_*7*_. The spectra show the signals of the corresponding atoms and point to chemical and isomeric purity of **3**.

The *cis*-[PtCl_2_(*n*aza)_2_] complexes containing *4Cl*aza (**1**), *3Br*aza (**2**), *4Br*aza (**3**), *3I5Br*aza (**4**), *3Cl5Br*aza (**5**) (Fig 1) were prepared by a simple synthetic strategy recently reported for the analogues involving different 7-azaindoles (*3Cl*aza (**I**), *3I*aza (**II**) and *5Br*aza (**III**); [19]). K_2_[PtCl_4_] was used as the starting platinum(II) compound directly reacting with a stoichiometric amount of the appropriate 7-azaindole derivative (*n*aza). The products were collected in *ca*. 80% yields and their chemical purity was determined by means of the results of elemental analysis and multinuclear NMR spectroscopy (see section Synthesis). The isomeric purity of the complexes **1**–**5**, as determined from the ratio of N1H signals of both the *cis*-[PtCl_2_(*n*aza)_2_] and *trans*-[PtCl_2_(*n*aza)_2_] isomers observed in the ^1^H NMR spectra, was found to be >95% (Fig 2).

The ESI+ mass spectrometry did not detect the molecular peaks of the studied complexes, but their adducts with the sodium or potassium ions were found according to their mass and isotopic distribution. The {*n*aza+H}^+^ fragments were observed in the spectra recorded in the positive ionization mode as well. The ESI–mass spectra contain the molecular peaks corresponding to the {[PtCl_2_(*n*aza)_2_]–H}^−^species (except for **5**) (S1 Fig). A release of one 7-azaindole-based ligand from the structure of the studied complexes led to the detection of the {[PtCl_2_(*n*aza)]–H}^−^and {*n*aza–H}^−^fragments within ESI–mass spectra of the studied complexes.

All the ^1^H, ^13^C and ^15^N signals of free 7-azaindole derivatives were found in appropriate spectra of the studied complexes. The anticipated N7-coordination mode of the 7-azaindole derivatives was unambiguously proved by the ^15^N coordination shifts (Δδ, ppm; calculated as Δδ = δ_complex_−δ_ligand_), whose values equal 2.5–3.6 ppm for N1 and −114.6–(–101.0) ppm for N7 (S1 Table). The atoms adjacent to the N7 coordination site shifted by 2.1–4.9 ppm downfield (for C6 in the ^13^C NMR spectrum), 0.5–2.0 ppm upfield (for C7a in the ^13^C NMR spectrum) and 0.52–0.99 ppm downfield (C6–H in the ^1^H NMR spectrum) (S1 Table). The ^195^Pt chemical shift values ranged from −2126 to −2113 ppm for **1**–**4**, while ^195^Pt NMR chemical shift for **5** was detected at −2183 ppm.

### Biological Activities Testing

#### *In vitro* cytotoxicity

The prepared complexes **1**–**5** and *cisplatin* (for comparative purposes) were tested by the commonly used MTT assay (*e*.*g*. [25]) for their *in vitro* antitumor activity against eight human cancer cell lines. The results are summarized in Table 1.

**Table 1:** The results of *in vitro* cytotoxicity of 1–5 and *cisplatin* (CDDP) against eight human cancer cell lines. Cells were treated with tested compounds for 24 h, measurements were performed in triplicate, and cytotoxicity experiment was repeated in three different cell passages. Data are expressed as IC_50_ ± SD (μM).

	1	2	3	4	5	CDDP
A2780	3.8±0.2*	4.1±0.4*	3.8±0.5*	4.8±1.0*	33.4±3.3	21.8±3.9
A2780R	3.6±0.7*	3.6±0.7*	3.5±1.1*	3.8±1.0*	24.7±1.0	32.0±9.6
HOS	7.5±2.4*	5.3±2.1*	4.5±2.7*	4.3±0.5*	46.7±4.0	25.4±8.5
G361	3.2±0.5	2.9±0.4	2.7±0.4	3.0±0.5	>50.0^a^	5.8±2.4
MCF7	3.5±1.0*	5.3±0.8*	2.7±1.2*	>10.0^a^	>50.0^a^	18.1±5.1
A549	11.6±4.2	19.0±5.6	11.1±0.3	>10.0^a^	>50.0^a^	>50.0^a^
HeLa	11.9±1.2*	17.1±0.8*	9.2±2.0*	>10.0^a^	>50.0^a^	39.9±4.6
LNCaP	3.9±0.1	4.9±0.1	4.0±0.6	>10.0^a^	>50.0^a^	3.8±1.5
RF^b^	0.95	0.88	0.92	0.79	0.74	1.47

asterisks (*), significantly different values (*p* < 0.05) between **1**–**5** and *cisplatin*

^a^) IC_50_ were not reached up to the given concentration

^b^) RF = resistance factor calculated as IC_50_(A2780R)/IC_50_(A2780)

**Fig 3:**
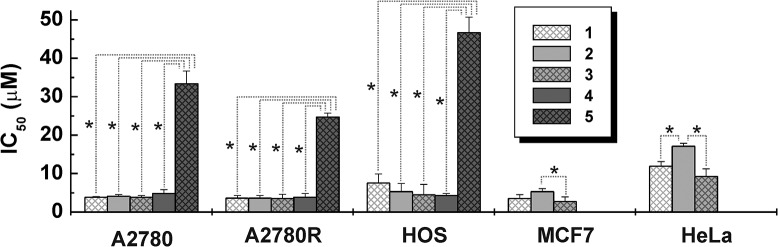
*In vitro* cytotoxicity of 1–5. Graphically depicted comparison of *in vitro* cytotoxicity of the studied complexes against ovarian carcinoma (A2780), *cisplatin*-resistant ovarian carcinoma (A2780R), osteosarcoma (HOS), breast carcinoma (MCF7) and cervix carcinoma (HeLa), where the significant differences (*p* < 0.05; assigned with the asterisks) between the obtained IC_50_ values (μM) of **1**–**5** were observed.

The IC_50_ values of **1**–**3** (involving monosubstituted 7-azaindole derivatives) against A2780, A2780R, HOS, G361, MCF7 and LNCaP were found to be lower than 7.5 μM (Table 1, Fig 3). *In vitro* cytotoxicity of **4** and **5** with disubstituted 7-azaindole derivatives is mutually different. **4** is, similarly to **1**–**3**, highly effective on the A2780, A2780R, HOS and G361 cancer cells (IC_50_ = 3.0–4.8 μM), while **5** is significantly (*p* < 0.05), *ca*. 1 order of magnitude less effective against A2780, A2780R and HOS (IC_50_ > 24.7 μM) as compared with **1**–**4** (Fig 3). The resistance factors (RF), calculated as a IC_50_(A2780R)/IC_50_(A2780) ratio, equalled 0.74–0.95 for **1**–**5** and 1.47 for *cisplatin* (Table 1). Further it has been found for the representative complex **3** that its *in vitro* cytotoxicity against A2780 increased from IC_50_ = 3.8±0.5 μM (without L-BSO) to IC_50_ = 2.6±0.1 μM with an addition of 5.0 μM L-buthionine sulfoximine (L-BSO), a well-known inhibitor of γ-glutamylcysteine synthetase.

#### Studies of stability and interactions with GSH and GMP

**Fig 4:**
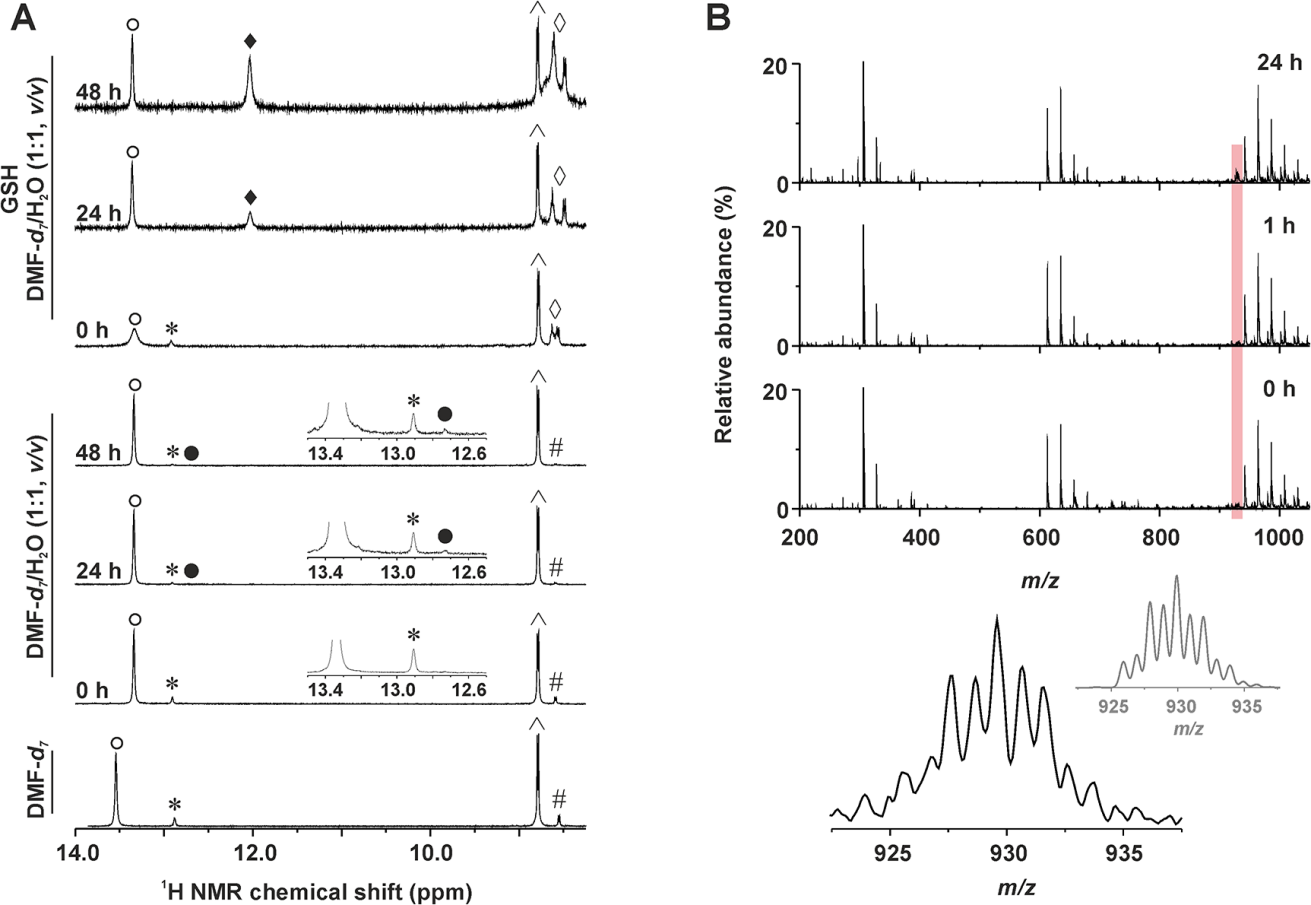
Time-dependent studies of stability of 3 (^1^H NMR) and its interaction with GSH (^1^H NMR and ESI-MS). (A) ^1^H NMR spectra of *cis*-[PtCl_2_(*4Br*aza)_2_] (**3**) dissolved in DMF-*d*_*7*_ or DMF-*d*_*7*_/H_2_O mixture (1:1, *v/*v) without or with glutathione (GSH) measured at different time points (0, 24 and 48 h). ○ = N1–H, *cis*-[PtCl_2_(*4Br*aza)_2_]; * = N1–H, *trans*-[PtCl_2_(*4Br*aza)_2_]; ● = N1–H, hydrolysis product, *cis*-[Pt(*4Br*aza)_2_(H_2_O)Cl]^+^; ♦ = N1–H, GSH adduct of **3**; ^ = C6–H, *cis*-[PtCl_2_(*4Br*aza)_2_]; # = C6–H, *trans*-[PtCl_2_(*4Br*aza)_2_]; ◊ = N–H of glycine and cysteine of GSH. (B) ESI–mass spectra (200–1050 *m/z* range) of the mixture of **3** with GSH (dissolved in the methanol/H_2_O, 1:1, *v/v*) as detected at different time points (0, 1 and 24 h). The region of the {[Pt(*4Br*aza)_2_Cl(GSH)]–2H}^−^species is highlighted by red colour. The detail of the experimental peak of {[Pt(*4Br*aza)_2_Cl(GSH)]–2H}^–^, as observed after 24 h, is given below (bottom left, given in black) together with the simulated isotope distribution (bottom right, given in grey).

The representative complexes **3** and **5** (dissolved in the DMF-*d*_*7*_/D_2_O mixture, 1:1 *v/v*) were studied by ^1^H NMR at different time points (0, 24 and 48 h) for its stability in the mentioned water-containing solvent. Except the signals (*e*.*g*. N1–H and C6–H) of **3** detected at 13.34 and 8.80 ppm (and *trans*-**3** impurity at 12.91 and 8.58 ppm), new signals rose in time at 12.70 and 8.45 ppm in connection with a formation of new species within the studied water-containing solution of **3** (Fig 4). The {[Pt(H_2_O)(*4Br*aza)_2_Cl]}^+^ species, whose intensity increased in time, was detected by ESI-MS experiments performed on **3** dissolved in the methanol/H_2_O mixture, 1:1 *v/v* (S2 Fig). In the case of **5**, any new N1–H peak was not detected in the ^1^H NMR spectra even after 48 h, however, its ability to hydrolyse (generally said, very low as in the case of **3**) can be anticipated based on the new C6–H signal, whose intensity increased in time (S3 Fig).

**Fig 5:**
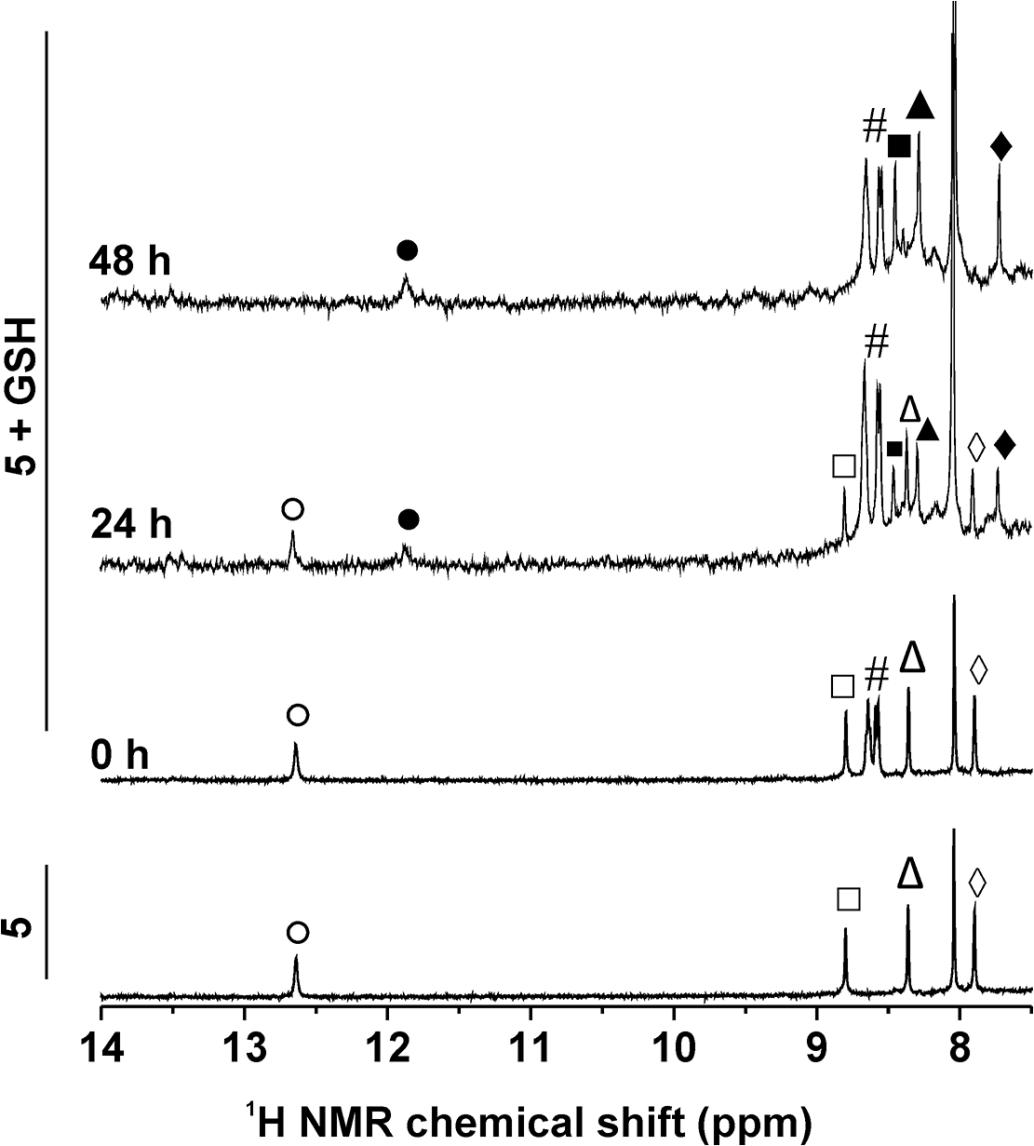
Time-dependent ^1^H NMR studies of 5 and its interaction with GSH. ^1^H NMR spectra of *cis*-[PtCl_2_(*3Cl5Br*aza)_2_] (**5**) dissolved in the DMF-*d*_*7*_/H_2_O mixture (1:1, *v/*v) without or with glutathione (GSH) measured at different time points (0, 24 and 48 h). ○ = N1–H, **5**; ● = N1–H, GSH adduct of **5**; □ = C6–H, **5**; ■ = C6–H, GSH adduct of **5**; Δ = C4–H, **5**; ▲ = C4–H, GSH adduct of **5**; ◊ = C2–H, **5**; ♦ = C2–H, GSH adduct of **5**; # = N–H of glycine and cysteine of GSH.

Analogical experiments (^1^H NMR, ESI-MS) were carried out for **3** and **5** with an addition of GSH. ^1^H NMR spectrum of **3** contained a new set of signals of the *4Br*aza ligand, represented by the N1–H signal at 12.03 ppm (Fig 3A). The ratio of the integral intensities of the N1–H signals of the starting complex and its analogue with GSH was approximately 1:1 after 48 h (*note*: an opacity observed in the solution after standing in the laboratory conditions belong to the *trans*-isomer of the starting complex, because its signals were not detected after centrifugation in the ^1^H NMR spectra after 24 and 48 h). A peak, whose mass and isotopic distribution corresponded to {[Pt(*4Br*aza)_2_Cl(GSH)]^+^} was detected by ESI-MS in the mixture of **3** and GSH (Fig 4). Complex **5** interacted with GSH as well, but *ca*. 1:1 integral intensity ratio of the N1–H signals of **5** (12.65 ppm) and its adduct with GSH (11.88 ppm) was reached after 24 h. After 48 h, the **5**+GSH spectrum contained only the signals of the adduct of **5** with GSH (Fig 5). The ^1^H NMR experiments were also performed on the mixtures of the studied complexes (**3** or **5**) with GMP. We did not observe any new *4Br*aza peaks in the spectra of **3** mixed with GMP even after 48 h (N1–H signal of **3** was not detected; S4 Fig). On the other hand, the spectrum of **5** contained several new peaks at 8.89, 8.23 and 7.74 ppm after 24 and 48 h of standing at laboratory temperature, belonging to C6–H, C4–H, and C2–H of the adduct of **5** with GMP, respectively (again, N1–H signal of **5** or its adduct with GMP was not detected; S5 Fig).

Opposing results obtained from the interaction experiments led us to investigation of behaviour of the studied complexes (**3** or **5**) mixed with both the used biomolecules, *i*.*e*. GSH and GMP. In the case of **3**+GSH/GMP, a ratio of the N1–H signals of **3** and its adduct with GSH was *ca*. 20:1 after 48 h (S4 Fig). Any new peaks, as compared with **3**+GSH and **3**+GMP experiments, were not detected in the **3**+GSH/GMP spectra. The results found for **5** showed that almost all the starting complex interacted with GSH after 48 h with the ratio of the N1–H signals of **5** and its GSH adduct being about 1:10 (S5 Fig). However, no signals of **5** and GMP adduct, as observed by the experiment without GSH addition (*i*.*e*. **5**+GMP, as discussed above), were detected in the spectra recorded on **5**+GSH/GMP.

#### Proteasome inhibition activity

**Fig 6:**
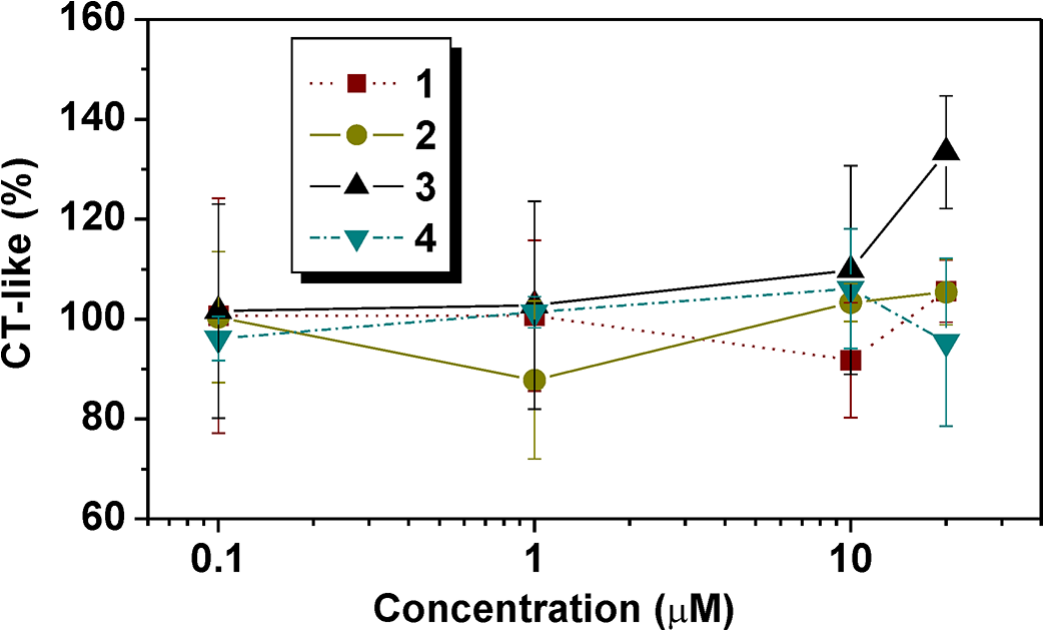
The results (CT-like activity (%±SD) of the studies of the ability of the complexes 1–4 to inhibit 20S proteasome activity assayed in purified proteasome obtained from A2780 cancer cell line

The ability of **1**–**4** to inhibit 20S proteasome activity, which was assayed in purified proteasome obtained from A2780 cancer cell line, was studied as well. The results are summarized in S2 Table and depicted in Fig 6. Concentrations of the tested compounds were applied in decimal scale, up to 20 μM, due to the limitations in solubility. None of the compounds tested did significantly inhibit a catalytic activity of 20S proteasome, in the whole concentration range.

## Discussion

The studied *cis*-[PtCl_2_(*n*aza)_2_] complexes (**1**–**5**; Fig 1) were prepared in good yields and high chemical and isomeric purity (as proved by ^1^H NMR studies; Fig 2) by one-step reactions directly from K_2_[PtCl_4_], as recently reported for the analogues with different 7-azaindoles [19,26]. The composition of **1**–**5** was proved by elemental analysis, ESI-MS spectrometry (S1 Fig) and multinuclear NMR spectroscopy. Since this work lacks results of a single crystal X-ray analysis (crystals suitable for X-ray analysis were not obtained yet), NMR spectroscopy was crucial technique for this work in terms of the structure description of the studied complexes **1**–**5**. Previously, an *N*7-coordination mode of the 7-azaindole (aza) and its halogeno-derivatives (*e*.*g*. *3Cl*aza) was proved by a single crystal X-ray analysis for *cis*-[PtCl_2_(aza)_2_] [26] and *cis*-[PtCl_2_(*3Cl*aza)_2_] [19]. Moreover, these complexes showed considerable difference between the ^15^N NMR coordination shifts (Δδ) of both the nitrogen atoms involved within the 7-azaindole moiety, in particular 2.3 ppm (N1) vs. −102.8 ppm (N7) for *cis*-[PtCl_2_(aza)_2_] [26], and 2.7 ppm (N1) vs. −101.5 ppm (N7) for *cis*-[PtCl_2_(*3Cl*aza)_2_] [19]. In the case of herein reported complexes **1**–**5**, the same conclusion regarding a coordination mode of the used 7-azaindoles can be done based on the obtained ^15^N NMR results (Experimental section and S1 Table). The changes in the electron density distribution, caused by the coordination of the 7-azaindole derivatives to the central Pt(II) atom through the N7 atom, led besides high Δδs of the N7 atoms also to the typical changes in the ^1^H and ^13^C chemical shift values, especially those ones adjacent to the N7 coordination site (S1 Table). The ^195^Pt NMR chemical shifts of **1**–**4** are in good agreement with recently reported *cis*-dichloridoplatinum(II) complexes involving 7-azaindole (–2120 ppm) [26] or its differently substituted halogeno-derivatives (from −2112 to −2126 ppm) [19]. Interestingly, ^195^Pt NMR chemical shifts differ between **1**–**4** and **5** by *ca*. 60 ppm, which has to be highlighted here with respect to the described differences in the *in vitro* cytotoxicity results obtained for **1**–**4** in comparison to **5**.

Regarding *in vitro* cytotoxicity of the studied complexes **1**–**5**, it has to be emphasized, that **1**–**3** (involving monosubstituted 7-azaindole derivatives) and **4** and **5** (involving disubstituted 7-azaindole derivatives) differ in the degree of substitution (Fig 1). In the case of **1**–**3**, their cytotoxic effect can be generalized as follows: these complexes showed acute *in vitro* cytotoxicity against A2780, A2780R, HOS, G361, MCF7 and LNCaP (Table 1). A comparison of the results obtained for **1**–**3** with *cisplatin* showed that all these substances are significantly more effective (*p* < 0.05) against A2780, A2780R, HOS, MCF7 and HeLa cancer cells than the mentioned clinically used platinum-based anticancer drug (Table 1). A quite different situation has to be discussed for the complexes **4** and **5** with disubstituted 7-azaindole derivatives, whose *in vitro* cytotoxicity is mutually different. Complex **4** is, similarly to **1**–**3**, highly effective against A2780, A2780R, HOS and G361, and in the case of A2780, A2780R and HOS cells even significantly more active as compared with *cisplatin* (*p* < 0.05; Table 1). A comparison of IC_50_ values of highly effective **1**–**4** emphasized that these substances did not differ in their *in vitro* cytotoxicity against A2780, A2780R, HOS, G361, A549 and LNCaP. In the case of MCF7 and HeLa cells, some differences were found. Particularly, IC_50_ of **3** is significantly lower as compared with **2** against breast cancer cells MCF7, and IC_50_ values of complexes **1** and **3** were found to be significantly lower than that of **2** against HeLa cell line (Fig 3).

Complex **4** showed lower solubility (to the 10.0 μM concentration only), and thus bioavailability, in the medium used (0.1% DMF), as compared with the other studied complexes **1**–**3** and **5**. Contrary to **4**, complex **5** was well-soluble (up to the upper tested concentration, *i*.*e*. 50.0 μM) but its IC_50_ values were significantly higher (*p* < 0.05) against A2780, A2780R and HOS as compared with **1**–**4**. Moreover, **5** was ineffective up to the upper tested concentration (IC_50_ > 50.0 μM) against G361, MCF7, A549, HeLa and LNCaP (Table 1). In other words, an implementation of the second substituent to the 7-azaindole moiety negatively affected either solubility (in the case of **4**) or cytotoxicity (in the case of **5**).

The well-established *in vitro* model of acquired *cisplatin-*resistance uses *cisplatin*-sensitive (A2780) and *cisplatin*-resistant (A2780R) ovarian carcinoma human cancer cell lines, numerically expressed as resistance factor (RF). The calculated RFs of **1**–**5** were considerably lower in comparison with *cisplatin* (Table 1). In other words, all the complexes **1**–**5** effectively overcome acquired *cisplatin*-resistance on the human ovarian carcinoma model.

Recently we reported the *in vitro* antitumor activity of three similar *cis*-dichloridoplatinum(II) complexes with halogeno-derivatives of 7-azaindole, namely *3Cl*aza (**I**), *3I*aza (**II**) and *5Br*aza (**III**), against eight cancer cell lines used also in this work [19,21]. Comparison of their *in vitro* cytotoxic activity of all eight *cis*-dichloridoplatinum(II) complexes with halogeno-derivatives of 7-azaindole (**1**–**5** and **I**–**III**) showed previously reported **III** as the highest active one with the IC_50_ values being *ca*. 6.7-, 12.9-, 13.7-, 5.7-, 9.8-, 5.3- and 2.5-times lower than those of *cisplatin* against A2780, A2780R, HOS, G361, MCF7, A549 and LNCaP, respectively. In the case of this work, the highest active complex **3** exhibited IC_50_ values about 5.7-, 9.1-, 5.6-, 2.2- and 6.7-times lower as compared with *cisplatin* against A2780, A2780R, HOS, G361 and MCF7, respectively. HeLa was the only cell line, where *in vitro* cytotoxicity of **III** (*ca*. 2.3-fold of *cisplatin*) was exceeded by its analogues, namely **1** (*ca*. 3.4-fold of *cisplatin*) and **3** (*ca*. 2.3-fold of *cisplatin*). It should be mentioned here as well that **III** showed the best and comparable with *cisplatin in vivo* results on mice from the tested complexes **I**–**III** [21]. Taken together all eight complexes with halogeno-derivatives of 7-azaindole (**1**–**5** and **I**–**III**), it can be concluded that the most pharmacologically perspective is position 5. From the structural point of view, this position is among the studied 3, 4 and 5 substituted derivatives the closest one to the coordination site of 7-azaindole ring (N7 atom; Fig 1). This phenomenon, that the distance of the substituent from the coordination site of the carrier ligand affects the biological properties of the complex involving such ligand, is known for *picoplatin* and its less active isomer involving 3-methylpyridine [15]. Other conclusions, which could be made based on the obtained results, is that: 1/ cytotoxicity is independent from the type and position of the substituent in the case of 3- and 4-substituted 7-azaindole derivatives, and 2/ the degree of the substitution could be more decisive than its type and position.

Interactions of the cytotoxic platinum(II) complexes, such as **1**–**5** or the clinically used *cisplatin*, with sulphur-containing biomolecule GSH is known to be one of the crucial mechanistic steps of their action [3,27]. It is connected with intracellular transport of the platinum(II) species to the target DNA molecule and, more importantly, with their inactivation and eflux from the cancer cells, known as one of the mechanisms of the cancer cell resistance. Because it has been found that the studied complexes, represented by **3** and **5**, interact with GSH, it was of the great interest to evaluate, whether the *in vitro* cytotoxicity of the studied complexes can be modulated by reduction of the intracellular GSH level by co-application of **3** (selected as a representative complex) with an inhibitor of γ-glutamylcysteine synthetase L-BSO. L-BSO has a direct effect on the synthesis of GSH and its application reduces the cellular level of GSH. The reduction of the GSH level should lead to higher cytotoxicity of such compounds, which are effectively inactivated by GSH [28] or whose mechanism of action is connected with the redox processes [29]. It has been found for **3** that its *in vitro* cytotoxicity against A2780 increased about 1.5-times. This observation is very important for further studies of herein reported complexes (*e*.*g*. *in vivo* studies of their anticancer activity), because it indicated that co-administration of these complexes with L-BSO can improve their biological effect against the cancer cells, as previously proved for platinum-based chemotherapeutics *cisplatin* and *oxaliplatin* [30,31].

The time-dependent ^1^H NMR and ESI-MS studies of stability of the representative complexes **3** and **5** dissolved in the water-containing solvent (DMF-*d*_*7*_/D_2_O mixture, 1:1 *v/v*) indicated very low ability of **3** and **5** to hydrolyse. Regarding highly *in vitro* cytotoxic complex **3**, new set of signals was detected in the ^1^H NMR spectra recorded 24 and 48 h after the preparation of the solution, as compared to the fresh solution (Fig 4). Since the chemical shifts of the new signals do not correspond to those of *cis*-[PtCl_2_(*4Br*aza)_2_], *trans*-[PtCl_2_(*4Br*aza)_2_] or the free *4Br*aza molecule, it can be concluded, that they belong to the new platinum-containing species, most probably to the *cis*-[Pt(*4Br*aza)_2_(H_2_O)Cl]^+^ or *cis*-[Pt(H_2_O)_2_(*4Br*aza)_2_]^2+^ or products of their protolytic reactions. To get evidence regarding the composition of new species, we performed the ESI-MS experiments on **3** dissolved in the methanol/H_2_O mixture, 1:1 *v/v*. We detected a peak of {[Pt(H_2_O)(*4Br*aza)_2_Cl]}^+^ with an intensity increasing in time (S2 Fig). As for **5**, whose *in vitro* cytotoxicity was considerably lower as compared with **3**, its very low ability to hydrolyse was proved by analogical ^1^H NMR studies (S3 Fig).

The results of ^1^H NMR clearly indicated that **3** gradually interacted with GSH with *ca*. 1:1 ratio of the integral intensities of the N1–H signals after 48 h observed for the starting complex **3** and its adduct with GSH, whose composition, as suggested by ESI-MS experiments, corresponds to {[Pt(*4Br*aza)_2_Cl(GSH)]^+^} (Fig 4). Interestingly, **5** interacted with GSH markedly faster than **3**, which could be suggested as one of the reasons connected with significantly lower antiproliferative effect of **5** as compared with **1**–**4** (represented by **3** within the stability and interaction studies) (Fig 5), because it is well-known fact that platinum complexes interact with GSH and other sulphur-containing biomolecules, which is connected with their inactivation [3,6,32].

Different reactivity between **3** and **5** towards another employed biomolecule (GMP) was also judged by the ^1^H NMR experiments. Concretely, no reactivity of highly cytotoxic complex **3** towards GMP was detected by ^1^H NMR (S4 Fig), while moderately active **5** interacted with the mentioned biomolecule, as proved by formation of new peaks of *3Cl5Br*aza detected in the spectra (S5 Fig). This finding is very interesting from the mechanistic point of view, because it is well-known that interaction of the *in vitro* cytotoxic platinum(II) complexes, and especially *cisplatin* analogues such as herein reported *cis*-dichloridoplatinum(II) complexes **1**–**5**, with nucleobases is crucial from the mechanistic point of view, because activity of such substances is based on their covalent interaction with nuclear DNA. Moreover, an ability of similar complexes with differently substituted 7-azaindoles to platinate nuclear DNA was clearly proved in one of our previous works [20].

With respect to the observations that **5** interacts with GMP, interacts faster than **3** with GSH and has lower *in vitro* cytotoxicity than **3**, it was of great interest to investigate behaviour of **3** and **5** in the presence of the GSH/GMP mixture. These experiments could show which biomolecule binds preferentially to the studied complexes and whether one biomolecule can replace another one within the inner coordination sphere on the pharmacologically relevant timescale (48 h). It was found that the ratio of the N1–H signals of **3** and its adduct with GSH differs markedly after 48 h with (approximately 20:1) or without (1:1) addition of GMP, which means that the presence of GMP affects either the process of interaction of **3** with GSH or the stability of the GSH adduct with **3** (S4 Fig). Further, any new peaks, as compared with **3**+GSH and **3**+GMP experiments, were not detected in the **3**+GSH/GMP spectra. In other words an interaction of the complex **3** with GMP was not observed even by this ^1^H NMR experiments. In the case of **5**, an interaction of this complex with GMP (as observed by **5**+GMP experiments) was suppressed by an addition of GSH, because only the peaks of the adduct of **5** and GSH were detected in the **5**+GSH/GMP spectra after 48 h (S5 Fig).

Taken together, the results of interaction experiments could prove that 1) affinities of GSH and GMP to **5** are higher as compared with **3**; 2) rate of interaction with GSH (or stability of the GSH adducts) is reduced by an addition of GMP in the case of **3**, which is not the case of **5**; 3) an interaction of **5** and GMP is suppressed in the presence of GSH.

The results of the mechanistic studies performed on the analogues (**I**–**III**) of the herein studied complexes **1**–**5** were discussed in our previous papers [19,20]. Therein reported complexes showed the *cisplatin*-like mechanism of action with some differences from *cisplatin*, such as higher cell-uptake and DNA platination. In an effort to bring in new mechanistic knowledge about the *cis*-dichloridoplatinum(II) complexes with 7-azaindole derivatives, we studied the ability of **1**–**4** to inhibit 20S proteasome activity assayed in purified proteasome from A2780, as described in the Experimental section (S2 Table, Fig 6). However, any inhibition of 20S proteasome was not observed for the studied complexes **1**–**4**.

To conclude this work, *cis*-dichloridoplatinum(II) complexes involving 7-azaindole derivatives were studied for their *in vitro* cytotoxicity against a panel of eight human cancer cell lines. Complexes **1**–**3** with monosubstituted *N*-donor ligands showed significantly (*p* < 0.05) higher *in vitro* cytotoxic effect against A2780, A2780R, HOS, MCF7 and HeLa as compared with *cisplatin*, with the IC_50_ values of **1**–**3** towards the mentioned cell lines ranging from 2.7 to 17.1 μM. An insertion of the second halogeno-substituent on the 7-azaindole moiety led to either lower solubility (**4**) or lower biological effect (**5**) of such complexes. The obtained results also indicate the ability of **1**–**5** to overcome intrinsic *cisplatin*-resistance on the ovarian carcinoma model. The ability of the complexes to hydrolyse in the water-containing solutions was found to be quite limited. Complex **3** interacted with GSH, but did not interact with GMP. The rate of interaction of **3** with GSH was markedly reduced with an addition of GMP. In the case of **5**, which interacted with both used biomolecules (GSH, GMP), it has been found that this complex prefers GSH during the interaction experiment with the mixture of GSH and GMP. Because the mechanistic studies on the analogues with differently substituted 7-azaindole derivatives were recently reported, we did not perform them on the herein reported complexes. However, in an effort to bring in new mechanistic finding regarding the *cis*-dichloridoplatinum(II) complexes with 7-azaindoles, we performed a study of their ability to inhibit the activity on the 20S proteasome from the A2780 cells, because the proteasome represents a highly promising target for anticancer drugs [33], but we did not observed any inhibition effect. This work, together with our previous papers dealing with *cis*-dichloridoplatinum(II) complexes containing 7-azaindoles, indicated that the complexes with 5-substituted derivatives of 7-azaindole exceed *in vitro* cytotoxicity of the complexes with the derivatives substituted at the positions 3 or 4. The discussed observations are quite challenging for the future work on the platinum complexes involving 7-azaindole derivatives with different 5-substituted derivatives and newly with the preparation and biological evaluation of hopefully even more pharmacologically perspective 6-substituted derivatives of 7-azaindole with the mentioned heterocyclic compound substituted next to its coordination site. Regarding the future biological experiments, the selected representatives of the dichloridoplatinum(II) complexes containing halogeno-derivatives of 7-azaindole, including those reported in this work, should be studied for their potency in the National Cancer Institute (NCI) NCI-60 cell-line screen, known to predict the mechanisms of action of the studied compounds. Moreover, the obtained NCI-60 results should also mechanistically distinguish the studied compounds from the clinically used ones, such as *cisplatin* or *oxaliplatin*, which is one of the crucial requirements for the following *in vivo* experiments, and preclinical and clinical studies.

## Supporting Information

S1 Fig ESI–mass spectrum of 3. The spectrum (200–1050 *m/z* range) is given with the assigned (*) peek of the {[PtCl_2_(*3Br*aza)_2_]–H}^−^species, whose experimental and simulated isotopic distribution is inserted.
(TIF)

S2 Fig ESI+ mass spectra of 3 at different time points. ESI+ mass spectra (400–800 *m/z* range) of the solution of **3** in methanol/H_2_O mixture (1:1, *v/v*) at different time points (0, 2 and 12 h) showing the peaks of the {[Pt(*4Br*aza)_2_(H_2_O)Cl]}^+^ species (●), overlapped peaks of the {[Pt(*4Br*aza)_2_(CH_3_OH)Cl]}^+^ and {[PtCl_2_(*4Br*aza)_2_]+H}^+^ species (○), and overlapped peaks of the {[Pt^III^Cl_2_(*4Br*aza)_2_(H_2_O)]}^+^ and {[PtCl_2_(*4Br*aza)_2_]+Na}^+^ species (◊). The simulated mass spectrum with the above-mentioned species given in the ratio 4: 3: 1: 10: 8 is also depicted for comparative purposes (top). The experimental and simulated (red tringles) isotopic distribution of the {[Pt(*4Br*aza)_2_(H_2_O)Cl]}^+^ species is inserted on the right side.
(TIF)

S3 Fig Time-dependent ^1^H NMR spectra of 5. ^1^H NMR spectrum of **5** dissolved in the DMF-*d*_*7*_/H_2_O mixture (1:1, *v/*v) (up) and part of the time-dependent ^1^H NMR spectra of **5** dissolved in the DMF-*d*_*7*_/H_2_O mixture (1:1, *v/*v) measured at different time points (0, 24 and 48 h) (down).
(TIF)

S4 Fig ^1^H NMR studies on the interaction of 3 with GSH and GMP. ^1^H NMR spectra of **3** (bottom spectrum) dissolved in the DMF-*d*_*7*_/H_2_O mixture (1:1, *v/*v) with guanosine 5'-monophosphate disodium salt hydrate (GMP) or the GMP mixture with glutathione (GSH) measured at different time points (0, 24 and 48 h). ○ = N1–H, **3**; ● = N1–H, GSH adduct of **3**; □ = C6–H, **3**; ■ = C6–H, GSH adduct of **3**; Δ = C2–H, **3**; ▲ = C2–H, GSH adduct of **3**; ◊ = C5–H, **3**; ♦ = C5–H, GSH adduct of **3**; # = N–H of glycine and cysteine of GSH; * = C8–H of GMP.
(TIF)

S5 Fig ^1^H NMR studies on the interaction of 5 with GSH and GMP. ^1^H NMR spectra of **5** (bottom spectrum) dissolved in the DMF-*d*_*7*_/H_2_O mixture (1:1, *v/*v) with guanosine 5'-monophosphate disodium salt hydrate (GMP) or the GMP mixture with glutathione (GSH) measured at different time points (0, 24 and 48 h). ○ = N1–H, **5**; ● = N1–H, GSH adduct of **5**; □ = C6–H, **5**; ■ = C6–H, GSH adduct of **5**; ∼ = C6–H, GMP adduct of **5**; Δ = C4–H, **5**; ▲ = C4–H, GSH adduct of **5**; × = C4–H, GMP adduct of **5**; ◊ = C2–H, **5**; ♦ = C2–H, GSH adduct of **5**; ∧ = C2–H, GMP adduct of **5**; # = N–H of glycine and cysteine of GSH; * = C8–H of GMP.
(TIF)

S1 Table The ^1^H, ^13^C and ^15^N NMR coordination shifts (∆δ = δ_complex_−δ_ligand_; ppm) of 1–5. (PDF)

S2 Table The results of the studies of the ability of the tested compounds to inhibit 20S proteasome activity assayed in purified proteasome obtained from A2780 cancer cell line. (PDF)

S1 Text The results of FTIR and Raman spectroscopy. (PDF)
