# Supplementary figures and images for: Highly and Broad-Spectrum In Vitro Antitumor Active cis-Dichloridoplatinum(II) Complexes with 7-Azaindoles
Source: PLoS One. 2015 Aug 26;10(8):e0136338. doi: 10.1371/journal.pone.0136338 (PMC4550364; doi:10.1371/journal.pone.0136338)

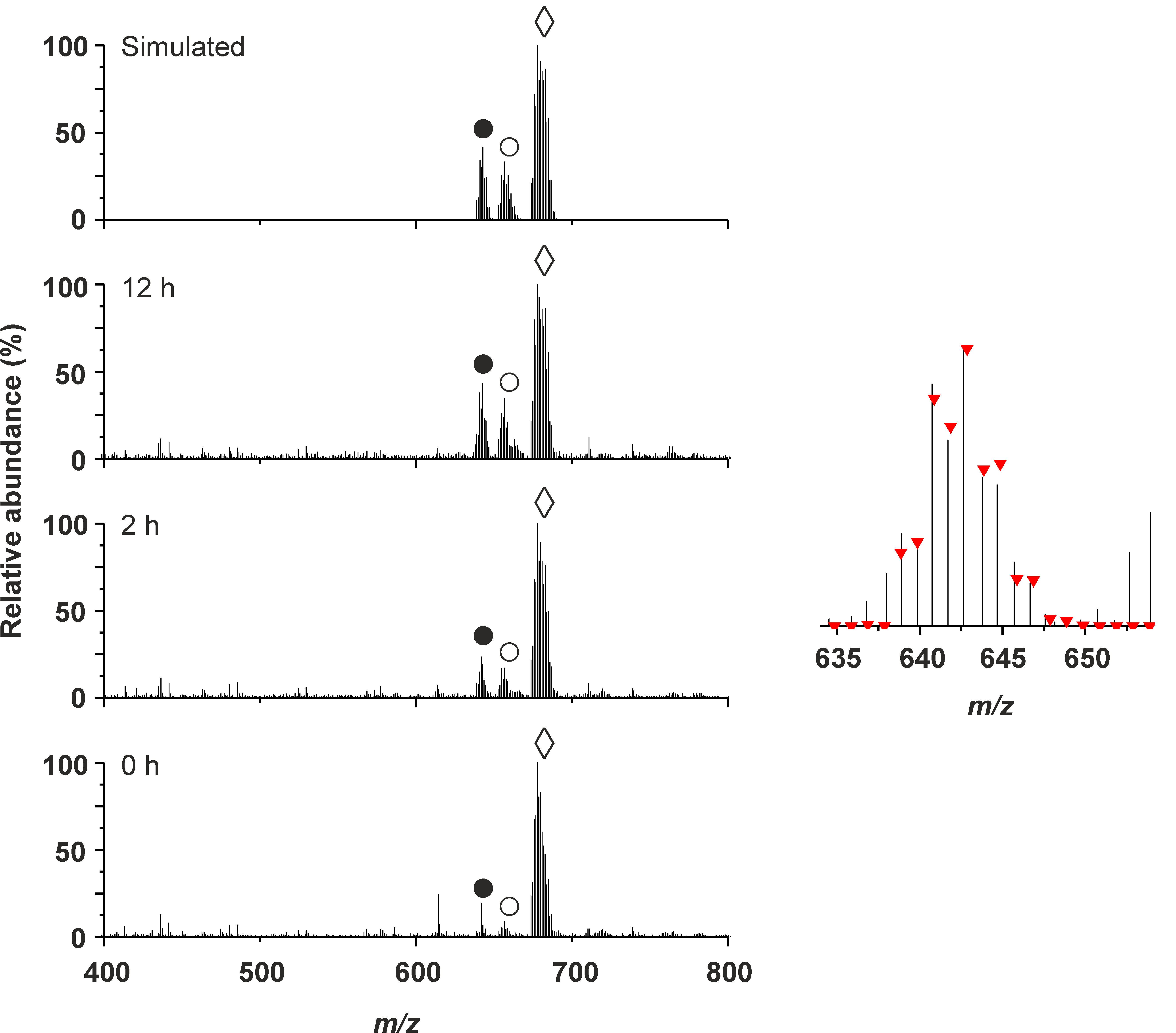

Supplement: S2 Fig — ESI+ mass spectra (400–800 m/z range) of the solution of 3 in methanol/H2O mixture (1:1, v/v) at different time points (0, 2 and 12 h) showing the peaks of the {[Pt(4Braza)2(H2O)Cl]}+ species (●), overlapped peaks of the {[Pt(4Braza)2(CH3OH)Cl]}+ and {[PtCl2(4Braza)2]+H}+ species (○), and overlapped peaks of the {[PtIIICl2(4Braza)2(H2O)]}+ and {[PtCl2(4Braza)2]+Na}+ species (◊). The simulated mass spectrum with the above-mentioned species given in the ratio 4: 3: 1: 10: 8 is also depicted for comparative purposes (top). The experimental and simulated (red tringles) isotopic distribution of the {[Pt(4Braza)2(H2O)Cl]}+ species is inserted on the right side. (TIF) [file pone.0136338.s002.tif]

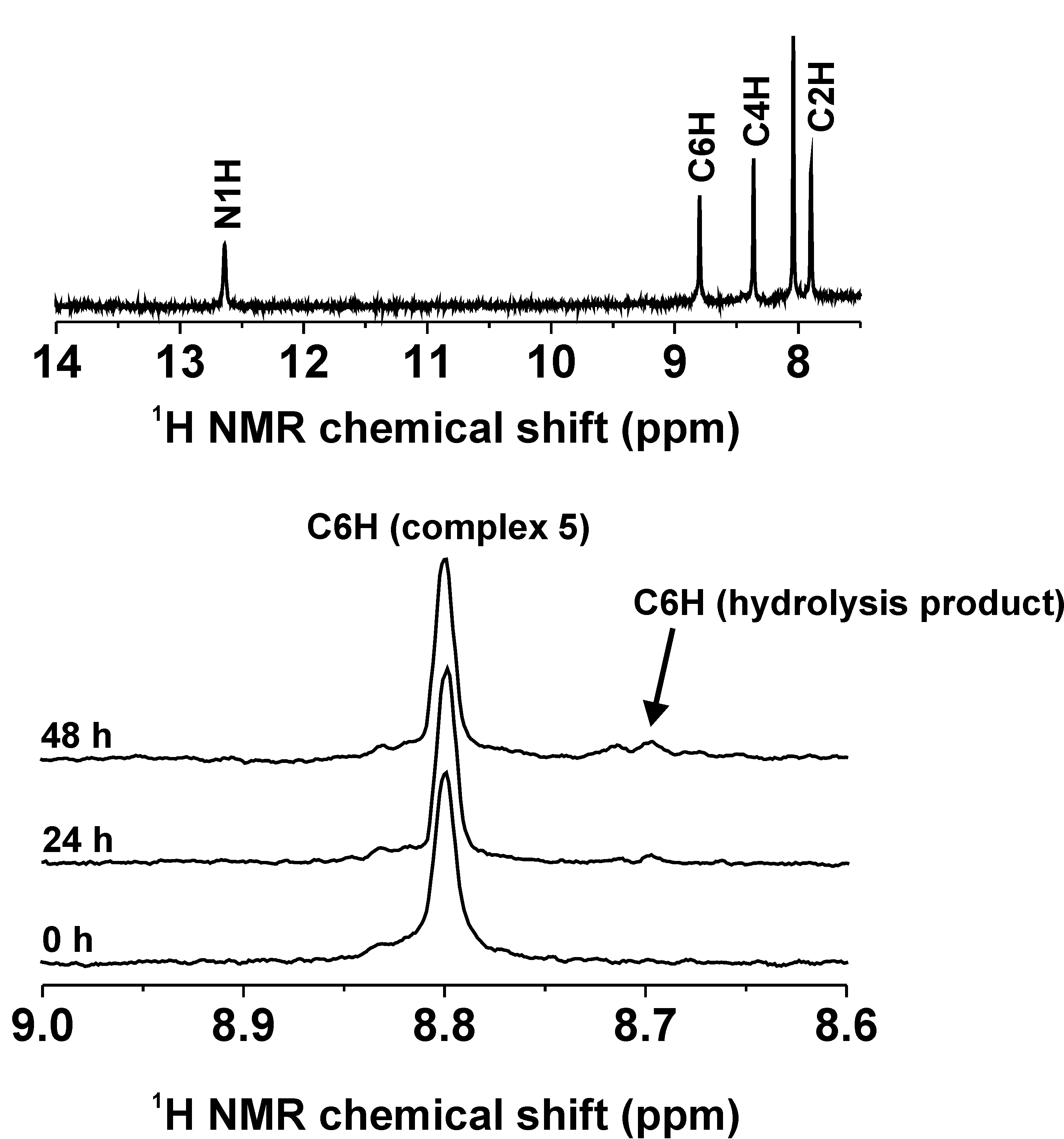

Supplement: S3 Fig — 1H NMR spectrum of 5 dissolved in the DMF-d 7/H2O mixture (1:1, v/v) (up) and part of the time-dependent 1H NMR spectra of 5 dissolved in the DMF-d 7/H2O mixture (1:1, v/v) measured at different time points (0, 24 and 48 h) (down). (TIF) [file pone.0136338.s003.tif]

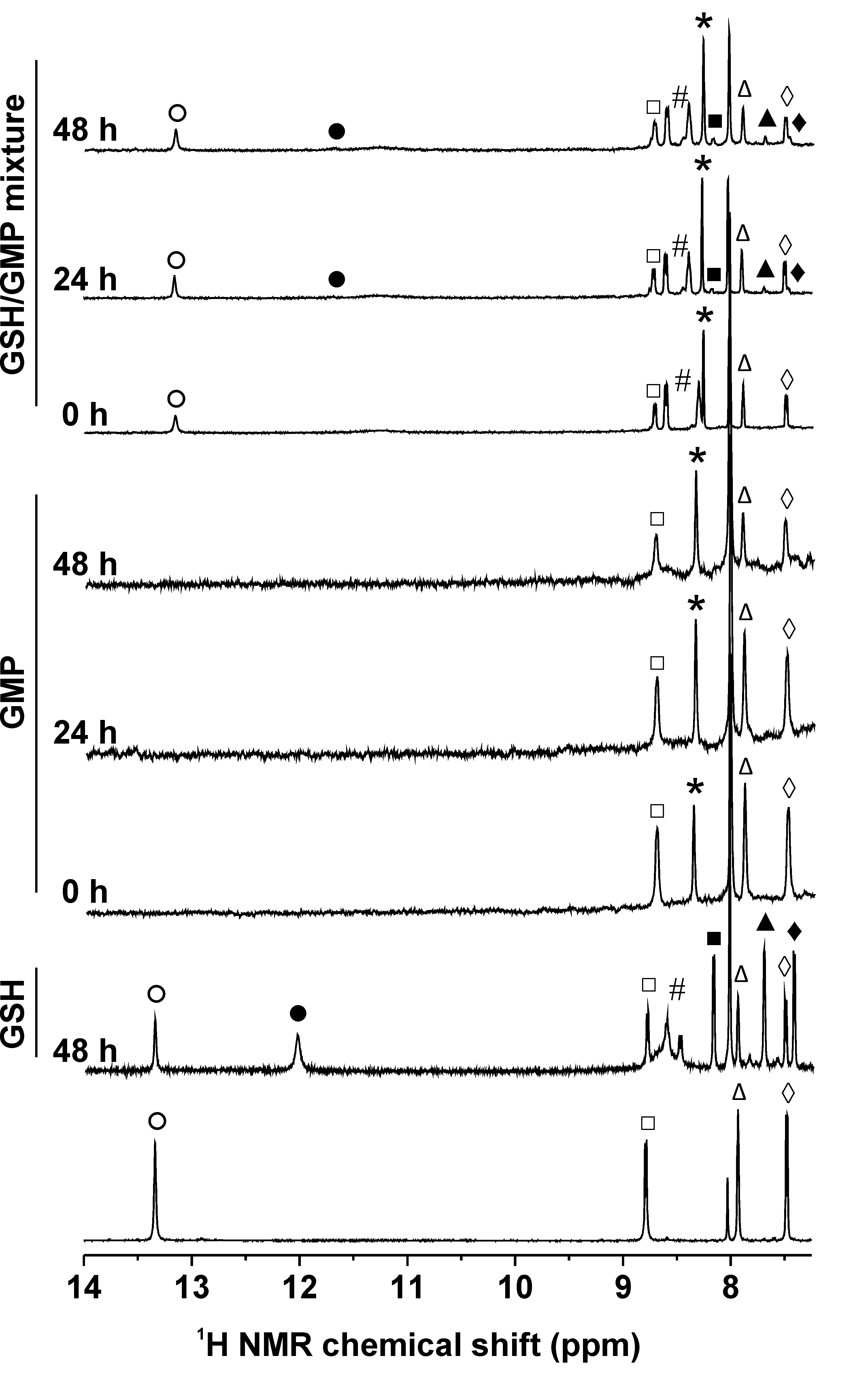

Supplement: S4 Fig — 1H NMR spectra of 3 (bottom spectrum) dissolved in the DMF-d 7/H2O mixture (1:1, v/v) with guanosine 5'-monophosphate disodium salt hydrate (GMP) or the GMP mixture with glutathione (GSH) measured at different time points (0, 24 and 48 h). ○ = N1–H, 3; ● = N1–H, GSH adduct of 3; □ = C6–H, 3; ■ = C6–H, GSH adduct of 3; Δ = C2–H, 3; ▲ = C2–H, GSH adduct of 3; ◊ = C5–H, 3; ♦ = C5–H, GSH adduct of 3; # = N–H of glycine and cysteine of GSH; * = C8–H of GMP. (TIF) [file pone.0136338.s004.tif]

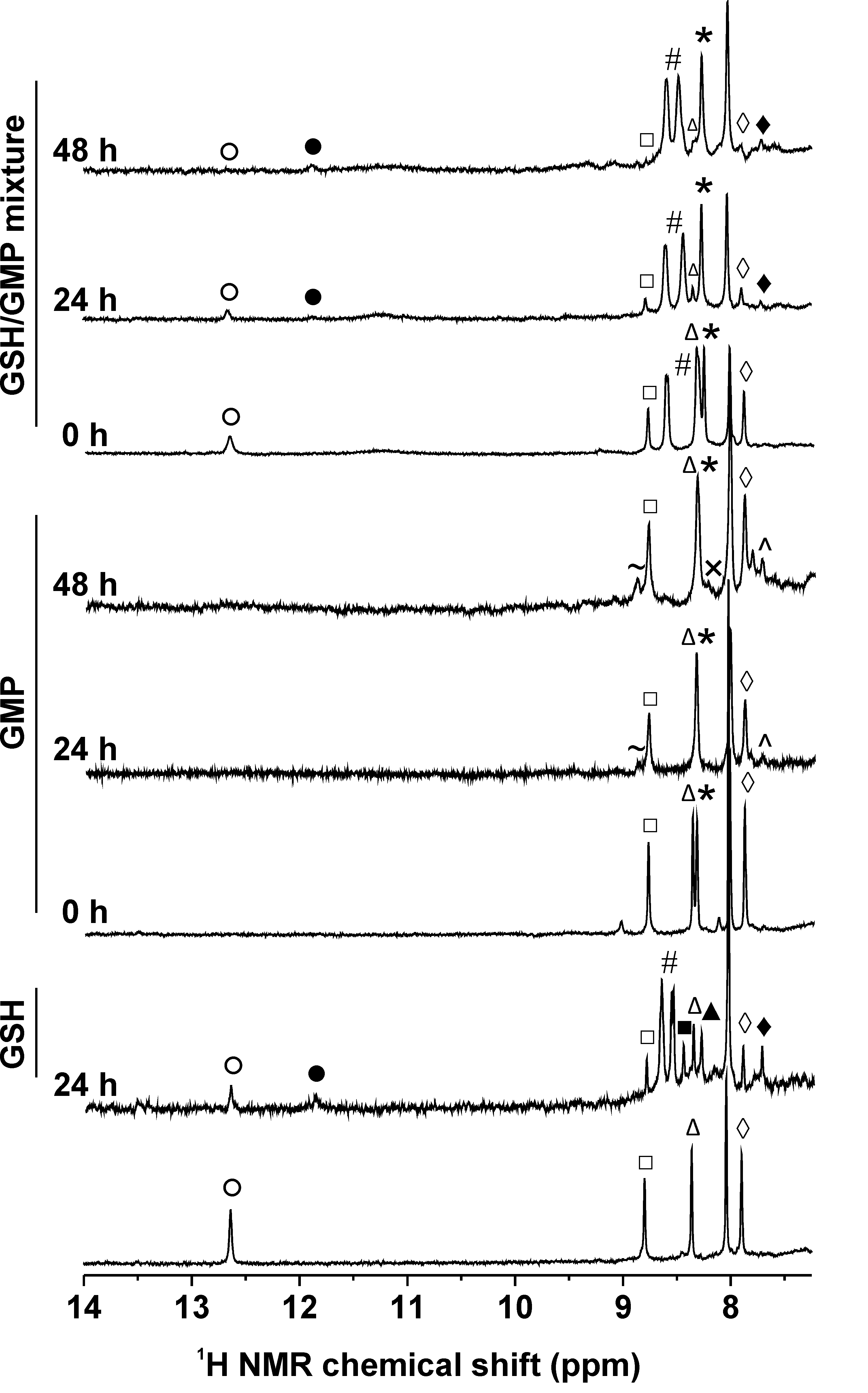

Supplement: S5 Fig — 1H NMR spectra of 5 (bottom spectrum) dissolved in the DMF-d 7/H2O mixture (1:1, v/v) with guanosine 5'-monophosphate disodium salt hydrate (GMP) or the GMP mixture with glutathione (GSH) measured at different time points (0, 24 and 48 h). ○ = N1–H, 5; ● = N1–H, GSH adduct of 5; □ = C6–H, 5; ■ = C6–H, GSH adduct of 5; ∼ = C6–H, GMP adduct of 5; Δ = C4–H, 5; ▲ = C4–H, GSH adduct of 5; × = C4–H, GMP adduct of 5; ◊ = C2–H, 5; ♦ = C2–H, GSH adduct of 5; ∧ = C2–H, GMP adduct of 5; # = N–H of glycine and cysteine of GSH; * = C8–H of GMP. (TIF) [file pone.0136338.s005.tif]
