# Supplementary material for: Highly and Broad-Spectrum In Vitro Antitumor Active cis-Dichloridoplatinum(II) Complexes with 7-Azaindoles
Source: PLoS One. 2015 Aug 26;10(8):e0136338. doi: 10.1371/journal.pone.0136338 (PMC4550364; doi:10.1371/journal.pone.0136338)
Supplement: S1 Table — (PDF) [file pone.0136338.s006.pdf]

The ^{1}H, ^{13} and ^{15}N NMR coordination shifts ($\Delta\delta = \delta_{complex} - \delta_{ligand}$; ppm) of **1–5**.

	^{1}H NMR						^{13}C NMR							^{15}N NMR	
	N1H	C2H	C3H	C4H	C5H	C6H	C2	C3	C3a	C4	C5	C6	C7a	N1	N7
**1**	1.45	0.26	0.09	–	0.12	0.66	1.9	1.9	2.9	3.1	1.6	2.2	-2.0	3.6	-101.2
**2**	1.37	0.32	–	0.08	0.09	0.68	3.3	1.4	3.2	1.7	1.3	2.5	-1.4	2.8	-101.7
**3**	1.44	0.26	0.09	–	0.11	0.66	1.5	2.0	3.3	3.0	1.6	2.1	-2.0	3.5	-101.0
**4**	1.45	0.11	–	0.27	–	0.99	3.5	2.0	2.6	1.3	-1.0	2.2	-0.5	2.5	-102.7
**5**	1.04	0.32	–	0.29	–	0.52	1.7	1.6	2.5	3.5	-1.5	4.9	-1.1	3.3	-114.6
