# Supplementary material for: Highly and Broad-Spectrum In Vitro Antitumor Active cis-Dichloridoplatinum(II) Complexes with 7-Azaindoles
Source: PLoS One. 2015 Aug 26;10(8):e0136338. doi: 10.1371/journal.pone.0136338 (PMC4550364; doi:10.1371/journal.pone.0136338)
Supplement: S2 Table — (PDF) [file pone.0136338.s007.pdf]

S2_Table: The results of the studies of the ability of the tested compounds to inhibit 20S proteasome activity assayed in purified proteasome obtained from A2780 cancer cell line.

**1** (µM)	CT- like activity (%)			***mean***	***SD***
0.1	125.3	78.5	98.2	**100.7**	*23.5*
1	118.1	92.1	91.9	**100.7**	*15.1*
10	102.9	79.9	92.7	**91.8**	*11.5*
20	106.7	111.1	98.9	**105.6**	*6.2*
**2** (µM)	CT- like activity (%)			***mean***	***SD***
0.1	100.7	87.2	113.4	**100.4**	13.1
1	100.6	70.2	92.7	**87.8**	15.8
10	101.1	107.7	101.2	**103.3**	3.8
20	112.3	104.6	99.3	**105.4**	6.5
**3** (µM)	CT- like activity (%)			***mean***	***SD***
0.1	86.6	126.1	92.1	**101.6**	*21.4*
1	90.3	126.9	91.3	**102.8**	*20.8*
10	98.7	133.9	96.8	**109.8**	*20.9*
20	124.4	146.1	129.6	**133.4**	*11.3*
**4** (µM)	CT- like activity (%)			***mean***	***SD***
0.1	100.2	96.8	91.4	**96.1**	*4.4*
1	100.8	104.8	98.6	**101.4**	*3.1*
10	106.4	117.9	93.9	**106.1**	*12.0*
20	83.2	114.6	88.5	**95.4**	*16.8*
