# Supplementary material for: Highly and Broad-Spectrum In Vitro Antitumor Active cis-Dichloridoplatinum(II) Complexes with 7-Azaindoles
Source: PLoS One. 2015 Aug 26;10(8):e0136338. doi: 10.1371/journal.pone.0136338 (PMC4550364; doi:10.1371/journal.pone.0136338)
Supplement: S1 Text — (PDF) [file pone.0136338.s008.pdf]

S1 Text. The results of FT-IR and Raman spectroscopies

cis-[$PtCl_2$(*4Cl*aza)$_2$] (**1**): IR (ν_{ATR}/cm^{-1}): 236m, 315vs, 323vs, 336vs, 434s, 491s, 510s, 530m, 542m, 560m, 569m, 579m. IR (ν_{ATR}/cm^{-1}): 439w, 511m, 543m, 605m, 656m, 722s, 781s, 809m, 826m, 861s, 921m, 953s, 1071m, 1113m, 1197vs, 1220m, 1257s, 1320vs, 1344vs, 1434s, 1487s, 1505s, 1587vs, 2747m, 2904m, 3097vs, 3164vs, 3201vs, 3418s, 3531s. Raman (cm^{-1}): 339m, 441w, 509w, 610s, 656m, 921w, 954w, 1072m, 1099s, 1257m, 1314m, 1344w, 1425w, 1439w, 1485m, 1507vs, 1588m, 3092m, 3121m, 3138m.

cis-[$PtCl_2$(*3Br*aza)$_2$] (**2**): IR (ν_{ATR}/cm^{-1}): 253m, 281m, 303m, 322vs, 466m, 482s, 510s, 557m, 591m. IR (ν_{ATR}/cm^{-1}): 465w, 483m, 511m, 558m, 593m, 646m, 748s, 792s, 821w, 897w, 931w, 993s, 1059w, 1095s, 1128m, 1199s, 1237m, 1276vs, 1336vs, 1385w, 1442s, 1492s, 1514m, 1593s, 2835m, 2914s, 2942s, 3006s, 3099vs, 3138vs, 3175vs, 3212vs, 3408vs. Raman (cm^{-1}): 327vs, 599m, 650m, 803vs, 897m, 991m, 1058m, 1128m, 1192m, 1237m, 1275m, 1324s, 1409m, 1440m, 1492s, 1514vs, 1596m, 3057m, 3089m, 3134m.

cis-[$PtCl_2$(*4Br*aza)$_2$] (**3**): IR (ν_{ATR}/cm^{-1}): 238m, 317vs, 327vs, 343m, 367w, 450m, 501m, 524w, 536w, 558w, 577w. IR (ν_{ATR}/cm^{-1}): 453w, 503m, 537m, 570m, 600m, 643m, 721vs, 779s, 807s, 852s, 915m, 932m, 1068m, 1109m, 1185vs, 1218m, 1254vs, 1317vs, 1341vs, 1429s, 1480s, 1506s, 1584vs, 1625m, 2904m, 2993s, 3077vs, 3141vs, 3199vs, 3461s.

cis-[$PtCl_2$(*3I5Br*aza)$_2$] (**4**): IR (ν_{ATR}/cm^{-1}): 306m, 333vs, 410s, 424s, 454m, 490vs, 501s, 523vs, 535s, 542s, 552s, 582s. IR (ν_{ATR}/cm^{-1}): 418w, 488w, 521m, 552w, 596m, 634w, 710s, 746m, 780m, 833w, 884s, 902s, 994s, 1103s, 1185s, 1268vs, 1327vs, 1419m, 1468vs, 1493m, 1540w, 1580s, 1616s, 2822s, 2986vs, 3043vs, 3082vs, 3121vs, 3494s. Raman (cm^{-1}): 306s, 333m, 808vs, 904m, 996w, 1090w, 1184w, 1238m, 1279s, 1328w, 1417w, 1469w, 1495vs, 1581w, 3043w, 3086w, 3127w.

cis-[$PtCl_2$(*3Cl5Br*aza)$_2$] (**5**): IR (ν_{ATR}/cm^{-1}): 280m, 301s, 316vs, 333m, 443m, 482s, 523vs, 543m, 572s. IR (ν_{ATR}/cm^{-1}): 443w, 482m, 525s, 573s, 600m, 646w, 721s, 754m, 809s, 880s, 905s, 1027s, 1096s, 1196vs, 1275vs, 1333s, 1402m, 1428m, 1470vs, 1512m, 1585s, 1600m, 2986m, 3055s, 3082vs, 3105s, 3133s, 3302vs.

Table S1: The ^{1}H, ^{13} and ^{15}N NMR coordination shifts ($\Delta\delta = \delta_{complex} - \delta_{ligand}$; ppm) of the prepared complexes.

	^{1}H NMR						^{13}C NMR							^{15}N NMR	
	N1H	C2H	C3H	C4H	C5H	C6H	C2	C3	C3a	C4	C5	C6	C7a	N1	N7
**1**	1.45	0.26	0.09	–	0.12	0.66	1.9	1.9	2.9	3.1	1.6	2.2	-2.0	3.6	-101.2
**2**	1.37	0.32	–	0.08	0.09	0.68	3.3	1.4	3.2	1.7	1.3	2.5	-1.4	2.8	-101.7
**3**	1.44	0.26	0.09	–	0.11	0.66	1.5	2.0	3.3	3.0	1.6	2.1	-2.0	3.5	-101.0
**4**	1.45	0.11	–	0.27	–	0.99	3.5	2.0	2.6	1.3	-1.0	2.2	-0.5	2.5	-102.7
**5**	1.04	0.32	–	0.29	–	0.52	1.7	1.6	2.5	3.5	-1.5	4.9	-1.1	3.3	-114.6

Table S2: The results of the studies of the ability of the tested compounds to inhibit 20S proteasome activity assayed in purified proteasome obtained from A2780 cancer cell line.

**1** (μM)	CT- like activity (%)			***mean***	***SD***
0.1	125.3	78.5	98.2	**100.7**	*23.5*
1	118.1	92.1	91.9	**100.7**	*15.1*
10	102.9	79.9	92.7	**91.8**	*11.5*
20	106.7	111.1	98.9	**105.6**	*6.2*
**2** (μM)	CT- like activity (%)			***mean***	***SD***
0.1	100.7	87.2	113.4	**100.4**	13.1
1	100.6	70.2	92.7	**87.8**	15.8
10	101.1	107.7	101.2	**103.3**	3.8
20	112.3	104.6	99.3	**105.4**	6.5
**3** (μM)	CT- like activity (%)			***mean***	***SD***
0.1	86.6	126.1	92.1	**101.6**	*21.4*
1	90.3	126.9	91.3	**102.8**	*20.8*
10	98.7	133.9	96.8	**109.8**	*20.9*
20	124.4	146.1	129.6	**133.4**	*11.3*
**4** (μM)	CT- like activity (%)			***mean***	***SD***
0.1	100.2	96.8	91.4	**96.1**	*4.4*
1	100.8	104.8	98.6	**101.4**	*3.1*
10	106.4	117.9	93.9	**106.1**	*12.0*
20	83.2	114.6	88.5	**95.4**	*16.8*

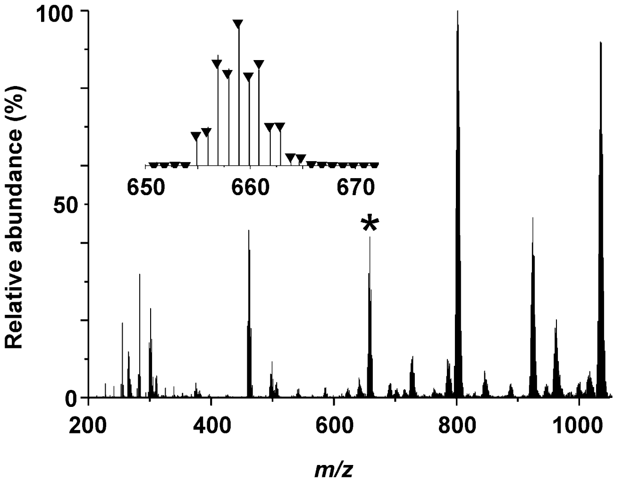

Figure S1: ESI– mass spectrum (200–1050 *m/z* range) of the methanolic solution of the complex **3** with assigned (*) peek of the {[$PtCl_2$(*3Br*aza)$_2$]–H}$^-$ species, whose experimental and simulated isotopic distribution is inserted

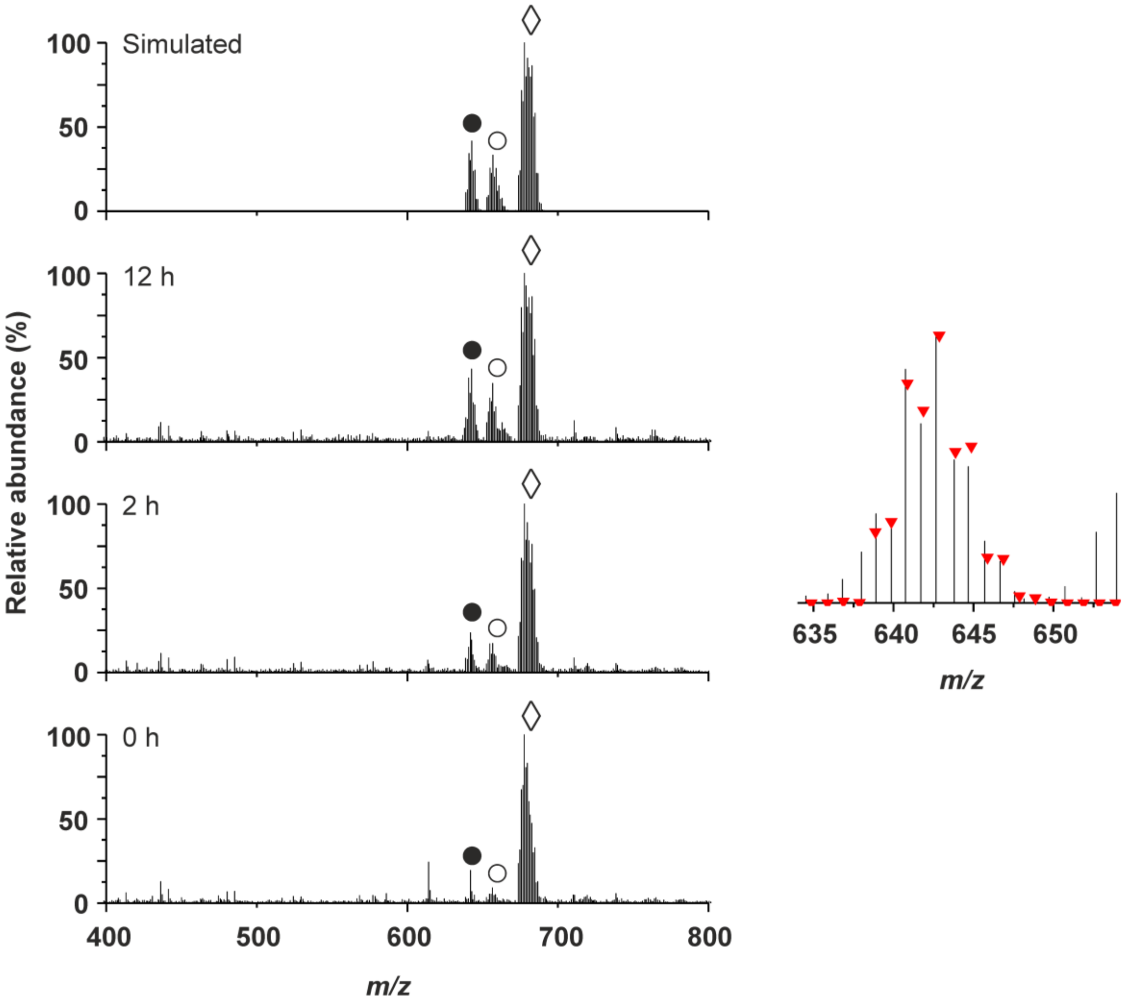

Figure S2: ESI+ mass spectra (400–800 *m/z* range) of the solution of the complex **3** in methanol/H_2O mixture (1:1, *v/v*) at different time points (0, 2 and 12 h). We detected the peaks of the {[Pt(*4Br*aza)$_2$(H_2O)Cl]}$^+$ species (●), overlapped peaks of the {[Pt(*4Br*aza)$_2$(CH_3OH)Cl]}$^+$ and {[PtCl$_2$(*4Br*aza)$_2$]+H}$^+$ species (○), and overlapped peaks of the {[Pt^{III}Cl$_2$(*4Br*aza)$_2$(H_2O)]}$^+$ and {[PtCl$_2$(*4Br*aza)$_2$]+Na}$^+$ species (◊). The simulated mass spectrum with the above-mentioned species given in the ratio 4 : 3 : 1 : 10 : 8 is also depicted for comparative purposes (top). The experimental and simulated (red tringles) isotopic distribution of the {[Pt(*4Br*aza)$_2$(H_2O)Cl]}$^+$ species is inserted on the right side.

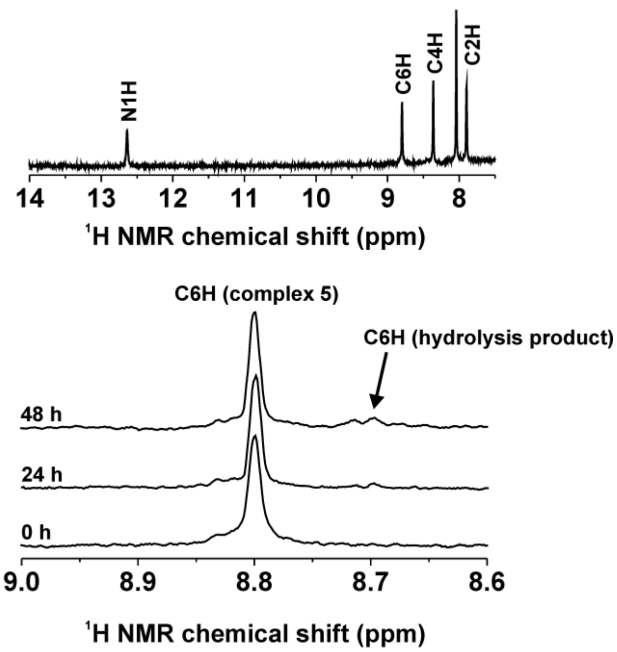

Figure S3: ^{1}H NMR spectrum of the complex *cis*-[$PtCl_2$(*3Cl5Br*aza)$_2$] (**5**) dissolved in the DMF-*d*$_7$/H_2O mixture (1:1, *v*/v) (up) and part of the time-dependent ^{1}H NMR spectra of **5** dissolved in the DMF-*d*$_7$/H_2O mixture (1:1, *v*/v) measured at different time points (0, 24 and 48 h) (down).

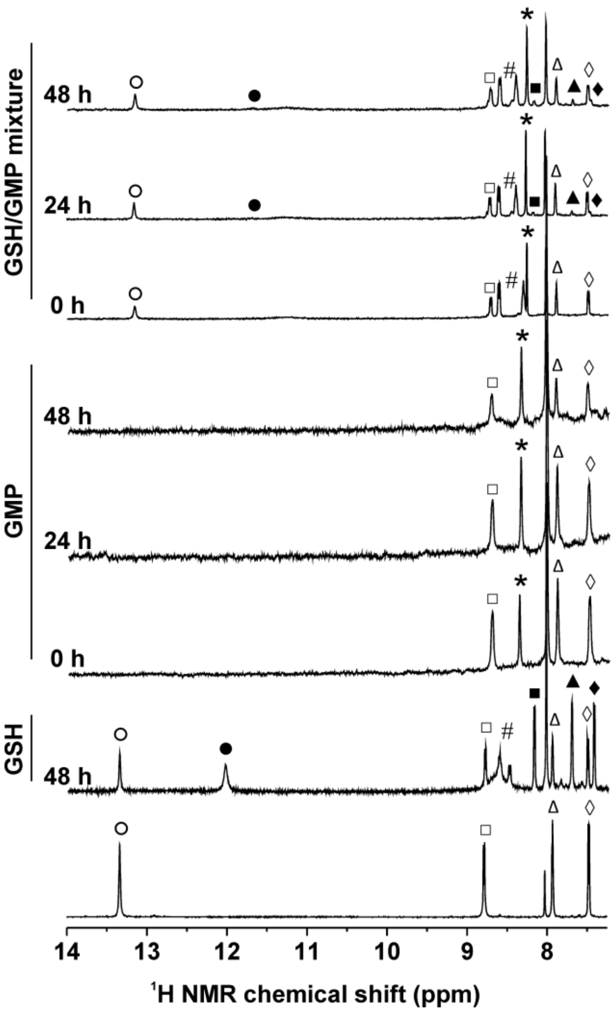

Figure S4: ^{1}H NMR spectra of the complex *cis*-[$PtCl_2$(*4Br*aza)$_2$] (**3**; bottom spectrum) dissolved in the DMF-d_7/H_2O mixture (1:1, *v/*v) with guanosine 5'-monophosphate disodium salt hydrate (GMP) or the GMP mixture with glutathione (GSH) measured at different time points (0, 24 and 48 h). ○ = N1–H, **3**; ● = N1–H, GSH adduct of **3**; □ = C6–H, **3**; ■ = C6–H, GSH adduct of **3**; Δ = C2–H, **3**; ▲ = C2–H, GSH adduct of **3**; ◊ = C5–H, **3**; ♦ = C5–H, GSH adduct of **3**; # = N–H of glycine and cysteine of GSH; * = C8–H of GMP.

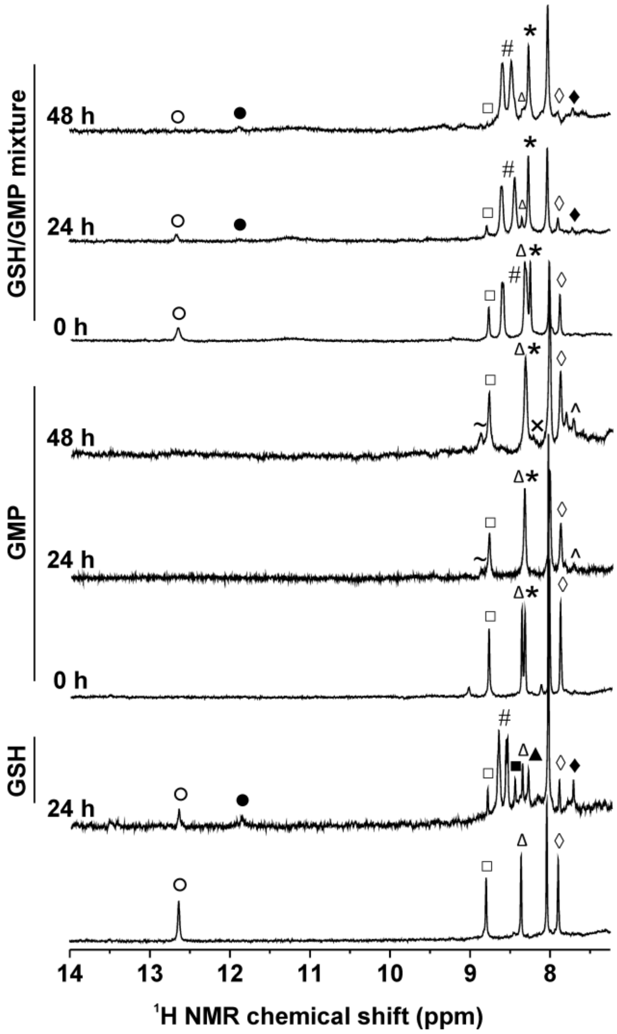

Figure S5: ^{1}H NMR spectra of the complex *cis*-[PtCl$_2$(*3Cl5Br*aza)$_2$] (**5**; bottom spectrum) dissolved in the DMF-d_7/H_2O mixture (1:1, *v/*v) with guanosine 5'-monophosphate disodium salt hydrate (GMP) or the GMP mixture with glutathione (GSH) measured at different time points (0, 24 and 48 h). ○ = N1–H, **5**; ● = N1–H, GSH adduct of **5**; □ = C6–H, **5**; ■ = C6–H, GSH adduct of **5**; ~ = C6–H, GMP adduct of **5**; Δ = C4–H, **5**; ▲ = C4–H, GSH adduct of **5**; × = C4–H, GMP adduct of **5**; ◊ = C2–H, **5**; ♦ = C2–H, GSH adduct of **5**; ∧ = C2–H, GMP adduct of **5**; # = N–H of glycine and cysteine of GSH; * = C8–H of GMP.
